# Immunomodulatory effects of tick saliva on dermal cells exposed to *Borrelia burgdorferi,* the agent of Lyme disease

**DOI:** 10.1186/s13071-016-1638-7

**Published:** 2016-07-08

**Authors:** Dorothy C. Scholl, Monica E. Embers, John R. Caskey, Deepak Kaushal, Thomas N. Mather, Wayne R. Buck, Lisa A. Morici, Mario T. Philipp

**Affiliations:** Divisions of Bacteriology and Parasitology, Tulane National Primate Research Center, Covington, LA USA; Center for Vector-Borne Disease, University of Rhode Island, Kingston, RI USA; Comparative Pathology, Tulane National Primate Research Center, Covington, Louisiana USA; Department of Microbiology and Immunology, Tulane University Medical School, New Orleans, LA USA; Present Address: Department of Biology, University of New Mexico, Albuquerque, NM USA; Present Address: AbbVie, 1 N Waukegan Rd, North Chicago, IL USA

**Keywords:** Monocyte/Macrophages, Fibroblasts, Lyme disease, *Borrelia burgdorferi*, Tick saliva, Cytokines, Chemokines, DNA microarray

## Abstract

**Background:**

The prolonged feeding process of ixodid ticks, in combination with bacterial transmission, should lead to a robust inflammatory response at the blood-feeding site. Yet, factors present in tick saliva may down-regulate such responses, which may be beneficial to spirochete transmission. The primary goal of this study was to test the hypothesis that tick saliva, in the context of *Borrelia burgdorferi*, can have widespread effects on the production of immune mediators in skin.

**Methods:**

A cross-section of tick feeding on skin was examined histologically. Human THP-1 cells stimulated with *B. burgdorferi* and grown in the presence or absence of tick saliva were examined by human DNA microarray, cytokine bead array, sandwich ELISA, and qRT-PCR. Similar experiments were also conducted using dermal fibroblasts.

**Results:**

Tick feeding on skin showed dermal infiltration of histiocytes and granulocytes at the bite location. Changes in monocytic transcript levels during co-culture with *B. burgdorferi* and saliva indicated that tick saliva had a suppressive effect on the expression of certain pro-inflammatory mediators, such as IL-8 (CXCL8) and TLR2, but had a stimulatory effect on specific molecules such as the Interleukin 10 receptor, alpha subunit (IL-10RA), a known mediator of the immunosuppressive signal of IL-10. Stimulated cell culture supernatants were analyzed via antigen-capture ELISA and cytokine bead array for inflammatory mediator production. Treatment of monocytes with saliva significantly reduced the expression of several key mediators including IL-6, IL-8 and TNF-alpha. Tick saliva had an opposite effect on dermal fibroblasts. Rather than inhibiting, saliva enhanced production of pro-inflammatory mediators, including IL-8 and IL-6 from these sentinel skin cells.

**Conclusions:**

The effects of ixodid tick saliva on resident skin cells is cell type-dependent. The response to both tick and pathogen at the site of feeding favors pathogen transmission, but may not be wholly suppressed by tick saliva.

**Electronic supplementary material:**

The online version of this article (doi:10.1186/s13071-016-1638-7) contains supplementary material, which is available to authorized users.

## Background

Vector-borne infectious diseases such as Lyme borreliosis (LB) result from complex interactions among three major components: host (mammal), pathogen (*Borrelia burgdorferi* (*sensu lato*)) and vector (*Ixodes* spp. ticks). *Borrelia burgdorferi*, the spirochete that causes LB, is transmitted during the feeding of infected *Ixodes* ticks*. Borrelia burgdorferi* lipoproteins and other pathogen-associated molecular patterns (PAMPs) are involved in the pathogenesis of LB by inducing the production of proinflammatory mediators in cells of the human host [1]. Yet, tick saliva contains several immunomodulatory factors that are thought to play a role in reducing or controlling the inflammatory response [2–4].

Characterizing the immunobiology of the tick-host interface is essential for understanding both tick feeding and pathogen transmission. It is well documented that saliva of blood-feeding arthropods enhances transmission of a variety of vector-borne disease-causing agents [5]. The current paradigm suggests that inhibition of host immune defenses via salivary components is a critical element in this process [6–8]. It has also been shown that events at the tick-host interface are so complex that successful transmission of pathogens often depends not only on the immunosuppression of host responses, but also on the enhancement of expression of certain vector genes [9, 10]. Furthermore, the enhancement of such genes is often mediated by the pathogen as exemplified by the *B. burgdorferi*-induced expression of two *Ixodes scapularis* genes, TROSPA (tick receptor for OspA) and Salp15 [9]. Salp 15 has been shown to bind to *B. burgdorferi* outer surface protein, OspC thereby inhibiting antibody-mediated killing of the pathogen. The establishment of *B. burgdorferi* infection, therefore, would not be possible without the enhanced expression of Salp15 by the tick.

Several pharmacological properties of tick saliva have been identified and include antihemostatic and vasoactive effects (Maxadilan and other vasodilators, such as the prostaglandin PGE_2_), the inhibition of complement, inactivation of anaphylatoxins and prevention of phagocytosis [2]. Other key immunomodulatory functions of arthropod saliva include inhibition of several cellular activities including nitric oxide production by macrophages [11], natural killer (NK) cell activity, the production of IFN-γ [12], histamine-binding capacity [13], and IgG-binding capacity [2, 14, 15]. It has been reported that tick saliva inhibits neutrophil function [16] and interferes with the complement system in vitro [17, 18]. Additional evidence suggests that chemical components of tick saliva modulate the host cytokine balance and shift cytokine production towards a Th2 response [18–20].

Upon delivery into the host, *B. burgdorferi* does not migrate away from the tick feeding site until several days after the tick has fed to repletion and has detached itself from the host [21, 22]. It is thought that the spirochetes do not migrate because the tick’s saliva conditions the host in a way that favors survival of the spirochetes early in infection [9]. Ixodid ticks require several days to feed to repletion. The hypothesis that in order to maintain feeding success, hard ticks require anti-inflammatory and immunosuppressive elements in their saliva has been proposed and supported by several studies [2, 6]. We specifically hypothesized that the saliva of the tick interferes with the innate immune response that is elicited by the spirochete in the dermal tissue at the initial site of blood feeding.

The following in vitro experiments were conducted in order to test our hypothesis regarding the effects of tick saliva on dermal cells: (i) the co-culture of activated human monocytes from the THP-1 cell line with *B. burgdorferi* in the presence and absence of tick saliva; and (ii) the co-culture of human or rhesus monkey fibroblasts with *B. burgdorferi* in the presence and absence of tick saliva. Cell culture supernatants from each experiment were used in ELISAs and/or multiplex cytokine bead arrays. RNA extracted from THP-1 monocytes at two time points during co-culture was used in microarray experiments. Validation of microarray results was gained by independent real-time PCR experiments. Here we demonstrate opposing effects of tick saliva on skin-resident cell types.

## Methods

### Imaging of tick attachment and feeding

A 4 mm biopsy was taken from a rabbit ear post-euthanasia to include the tick and site of feeding. This rabbit was used for saliva collection (partial engorgement of ticks). The biopsy was formalin-fixed, embedded in paraffin and sectioned at 6 μm. Sections were fixed to slides and stained with hematoxylin and eosin (H&E).

### Monocytes and fibroblasts

#### Monocytes

This study utilized the human monocytic cell line THP-1 (ATCC TIB-202). Differentiation was induced with Vitamin D-3, 1α, 25-Dihydroxy treatment (Calbiochem, San Diego, CA, USA) at a final concentration of 0.05 μM for a maximum of 72 h. This treatment is known to induce the expression of CD14 [23], macrophage morphology, macrophage-specific esterase enzyme activity, and phagocytosis [24]. Cells were seeded at an initial density of 5 × 10^5^/ml for pilocarpine control experiments and 2.5 × 10^5^ cells/ml for saliva experiments. Cells were stimulated with live *B. burgdorferi* strain B31-5A19 [25] (MOI 10:1) for 24 h in the presence or absence of pilocarpine (PC)-induced *I. scapularis* saliva, where a single dose [26] of 64 μg/ml of total salivary protein was added [27]. Controls included PC-only treated cells, as well as *B. burgdorferi* + PC- stimulated cells. After stimulation, cell viability was assessed via Trypan Blue staining, and found to be less than 10 % following the addition of saliva. Supernatants were harvested for cytokine bead array, and IL-6, IL-8 and TNF-α detection by antigen-capture ELISA.

#### Primary rhesus fibroblast purification and culture

The method for purifying and culturing primary rhesus fibroblasts used in this study was adapted from a previously published protocol used for human fibroblasts [28]. Skin biopsies were taken from animals that were not experimentally infected with pathogens and which were culled from the breeding colony because of chronic diarrhea or injury. The area for the skin biopsy was first shaved and then cleansed with Betadine and ethanol. Upon excision of the sample, it was immediately placed in 40 ml of cold Fibroblast Growth Medium 2 (FGM2, PromoCell, Heidleberg, Germany) and placed on ice. After rinsing with penicillin (100 UI/ml) and streptomycin (100 μg/ml) -supplemented sterile phosphate-buffered saline (PBS) (Sigma Chemical Company, St Louis, MO) the dermis was separated from the epidermis. Dermal fragments were then placed onto the scored surface of a sterile Petri dish, covered with FGM2, and allowed to adhere to the dish (approximately one h). Once the fragments were attached, 10 ml of FGM2, supplemented with penicillin (100 UI/ml) and streptomycin (100 μg/ml) and a proprietary supplement mix for FGM2 (PromoCell, Heidelberg, Germany) containing 2 % fetal calf serum, insulin (5 μg/ml) and basic fibroblast growth factor (1 ng/ml) was added. The dishes were then closed and placed in a 37°C incubator, under 5 % CO_2_. The culture medium was changed every two days in order to maintain an optimal pH range between 7.6 and 7.8. Fibroblast proliferation occurred in approximately 1–2 weeks. When cultures were approximately 80 % confluent, sub-culturing was done as described [28]. Fibroblasts were passaged three times prior to being used in experiments.

#### Human fibroblasts

Normal dermal human fibroblasts were obtained from the American Type Culture Collection (ATCC N° PCS-201-012). They were grown in PromoCell FGM 2, trypsinizsed with Promocell Trypsin/EDTA and neutralization solution as per the manufacturer’s instructions. For experiments, human fibroblasts were plated at 5 × 10^4^ cells/well.

### Induction of tick salivation

Saliva induction involved using a 1.0 % PC in methanol solution applied to the dorsal surface of a partially engorged adult female *I. scapularis* tick [29]. Collection of salivary secretions was accomplished via glass capillary tubes attached to the hypostome of each tick [30]. Salivary secretions were then pooled and protein concentration was measured using a nanodrop UV spectrophotometer, at 280 nm absorbance. Saliva was sterile-filtered through a 0.22 μm membrane prior to use. Contamination of the saliva with PC was quantified by high-performance liquid chromatography HPLC, as described later in this section.

### Determination of PC concentration in saliva samples by HPLC

#### Sample preparation and standard curve

Tick saliva (2–10 μl) was diluted with 200 μl of 10 mM sodium phosphate pH 7.0, 100 μof 1 mM caffeine (internal standard), and water to a final volume of 400 μl. The diluted sample was extracted with an equal volume of dichloromethane twice, and the organic phases were pooled and dried under nitrogen. The sample was reconstituted in 200 μl mobile phase A (see below), centrifuged briefly at 300 × g, and 20 μlwas injected with a Shimadzu SIL-10ADvp autosampler. Pilocarpine standards were prepared the same way as the samples. The standard concentrations were (in pmol/l) 2880, 1440, 720, 360, 180, 90 and 0.

#### Chromatography technique

Separation was performed using a ShimPack ODS column (4.6 × 15 mm, 5 μm particle size, 100 Angst pore size) at 30 °C. Mobile phase A was composed of 0.6 % triethylamine adjusted to pH 2.3 with phosphoric acid (85 % w/v); mobile phase B was methanol. Mobile phases were delivered by paired Shimadzu LC-10ADvp pumps at 1.5 ml/min. Pilocarpine was eluted with mobile phase A for 10 min. Subsequently, caffeine was eluted with a ratio of mobile phases A:B of 7:3 for 10 min. Then the column was equilibrated with mobile phase A for 10 min. Peaks were recorded with a Shimadzu SPD-10ADvp UV/Vis detector, set to 215 nm for pilocarpine and 275 nm for caffeine. The retention times for the pilocarpine and caffeine were 7.4 and 16.3 min, respectively.

#### Data analysis

Shimadzu EZStart 7.2.1 software was used for data analysis. Pilocarpine peak areas were compared against the internal standard, and logarithmically transformed peak area ratios were quantified against a linear regression standard curve. The concentration of pilocarpine in tick saliva was found to be 2.36 mM (2.39 pmol/μl).

### Determination of cytokine/chemokine concentrations by antigen-capture ELISA

THP-1 monocyte or primary fibroblast supernatants were collected at 12 and 24 h post-stimulation, aliquoted, and stored at -80 °C until used. Controls included supernatants from unstimulated cells. Supernatants were assessed for IL-6, IL-8 and TNF-α production by ELISA (R&D Systems) following the manufacturer’s instructions. Supernatants were assessed in triplicate and each assay was conducted twice. Statistical significance was determined by two-tailed *t* test.

### Cytokine bead arrays

THP-1 cell supernatants were collected after 2 and 12 h of stimulation. Controls included supernatants of unstimulated THP-1 cells. The human cytokine multiplex-27 (Luminex xMAP) bead array assay kit was obtained from BioRad Laboratories, Inc. (Hercules, CA) and used according to the manufacturer’s instructions. The following mediators were included: IL-1β, IL-1ra, IL-2, IL-4, IL-5, IL-6, IL-7, IL-8 (CXCL8), IL-9, IL-10, IL-12(p70), IL-13, IL-15, IL-17, Eotaxin, FGF basic, G-CSF, GM-CSF, IFN-γ, IP-10 (CXCL10), MCP-1 (CCL2), MIP-1α (CCL3), MIP-1β (CCL4), PDGF-BB, RANTES (CCL5), TNF-α, and VEGF. Supernatant samples were run in triplicate and analyzed with the Bio-plex 200 Suspension Array System (BioRad). The assay was performed twice. Each analyte concentration was calculated by logistic-5PL regression of the standard curve.

### Microarrays

RNA was isolated from approximately 2.5–5 × 10^5^ cells using the RNeasy kit (Qiagen, Valencia, CA, USA) and rendered free from DNA contamination using the DNA-free reagent (Ambion, Austin, TX). Five hundred ng RNA was amplified and used to synthesize Cy-labelled cDNA with the Amino Allyl Message Amp kit (Ambion, catalog No.1752). Human oligo microarray (44.5 K) chips obtained from the Stanford Functional Genomics Facility (Catalog No.SFGF101) were used for expression analyses in order to determine the levels at which certain genes are expressed with respect to control genes. Cy3 (control) and Cy5 (experimental) -labelled cDNA were mixed in equimolar quantities and hybridized overnight at 55 °C to SFGF whole-genome microarrays (4 × 44.5 K format), representing approximately 22,000 unique human genes. The slides were scanned on a GenePix 4000B scanner, and data were extracted from the resulting 16-bit TIFF images using GenePix Pro 6.1 software. Data were analyzed using Spotfire DecisionSite for Microarray Analysis. Values were log2 transformed and normalized using a Locally Weighted ScatterPlot Smoothing (LOWESS) script within S+ ArrayAnalyzer.

Analyses of microarrays were conducted using an approach that focused on specific genes related to immune responses. Within those genes were also a number of genes involved in apoptosis and cell cycle regulatory genes. Microarray data selected for analyses included only those genes that had a *P*-value of ≤ 0.05 and a change of ≥ 1.5-fold in both duplicate microarray experiments. Change and log ratio were calculated by combining the two duplicate microarray experiments to produce one value representing change (n-fold) and one value representing log ratio for each gene. One-way analysis of variance (ANOVA) was performed. An early time point of two hours post-stimulation was evaluated, as was a later time point of 12 h post-stimulation, in order to assess differences in response to *B. burgdorferi* and *B. burgdorferi* plus tick saliva. Data were analyzed through the use of Ingenuity Pathways Analysis (Ingenuity® Systems, www.ingenuity.com) and DAVID Bioinformatics Resources 6.7 (NIAID, NIH). Data sets from each time point and experimental treatment containing gene identifiers and corresponding expression values were uploaded into the application.

Each gene was mapped to its corresponding canonical pathway in Ingenuity’s Knowledge Base and with the DAVID database. A fold-change cut-off of 1.5 was set to identify genes whose expression was perturbed at each time point. The significance of the association between the data set and the canonical pathway was measured in two ways: (i) a ratio of the number of molecules from the data set that map to the pathway divided by the total number of molecules that map to the canonical pathway; (ii) Fisher’s exact test was used to calculate a *P*-value determining the probability that the association between the genes in the dataset and the canonical pathway is explained by chance alone.

Importantly, the perturbation of gene expression by saliva in the presence of *B. burgdorferi* must take into account the effect of *B. burgdorferi* alone. For example, if *B. burgdorferi* alone stimulates a 3-fold increase in expression of gene X, and the value for *B. burgdorferi* + tick saliva is -1.5 fold, the actual change in expression imparted by tick saliva is -4.5 fold. Therefore, from the 2 and 12-h time points, all genes that were differentially expressed in both groups (*B. burgdorferi* only and *B. burgdorferi* + saliva) were analyzed by subtracting the expression value of *B. burgdorferi* only from the value of *B. burgdorferi* + saliva. Only those genes that were significantly (1.5-fold or more) affected by both were included in this calculation. In order to further narrow the dataset to function relevant to the transmission of pathogen through the skin, those genes deemed related to immune responses by Ingenuity pathways were categorized into “immune function” or “apoptosis regulation” pathways using the DAVID database.

### Real-time PCR

Relative transcription profiles of IL-10RA and TLR2 were determined by qRT-PCR using the SYBR^®^ Green RT-PCR Assay kit (Applied Biosystems, Foster City, CA) with the respective primers specific for each. QuantiTect primers, which are validated, ready-to-use primer sets were obtained from Qiagen for each gene of interest. In addition, transcription of the housekeeping gene GAPDH was determined using specific primers. Real-time PCR reactions were set up in triplicate for each of the cytokines and the housekeeping gene. Amplification conditions were identical for all reactions and consisted of: 2 min at 50 °C, 10 min at 95 °C, 40 cycles of 15 s at 95 °C and 60 s at 60 °C. Reaction samples had a final volume of 25 μl consisting of 12.5 μl of Universal Master mix containing the specific primer/probe mix (Qiagen Quantitect Primer Assays) and 50 ng of the respective RNA template. Amplifications were run in an ABI Prism 7900 Sequence Detection System (Applied Biosystems). Analysis of each run was conducted utilizing the REST program for relative expression, which compares two groups based on PCR efficiencies and the mean crossing point deviation between the sample and the control group. The expression ratio results of the investigated transcripts were tested for significance by a randomization test [31].

## Results

### Skin damage and inflammation is evident during tick feeding

**Fig. 1:**
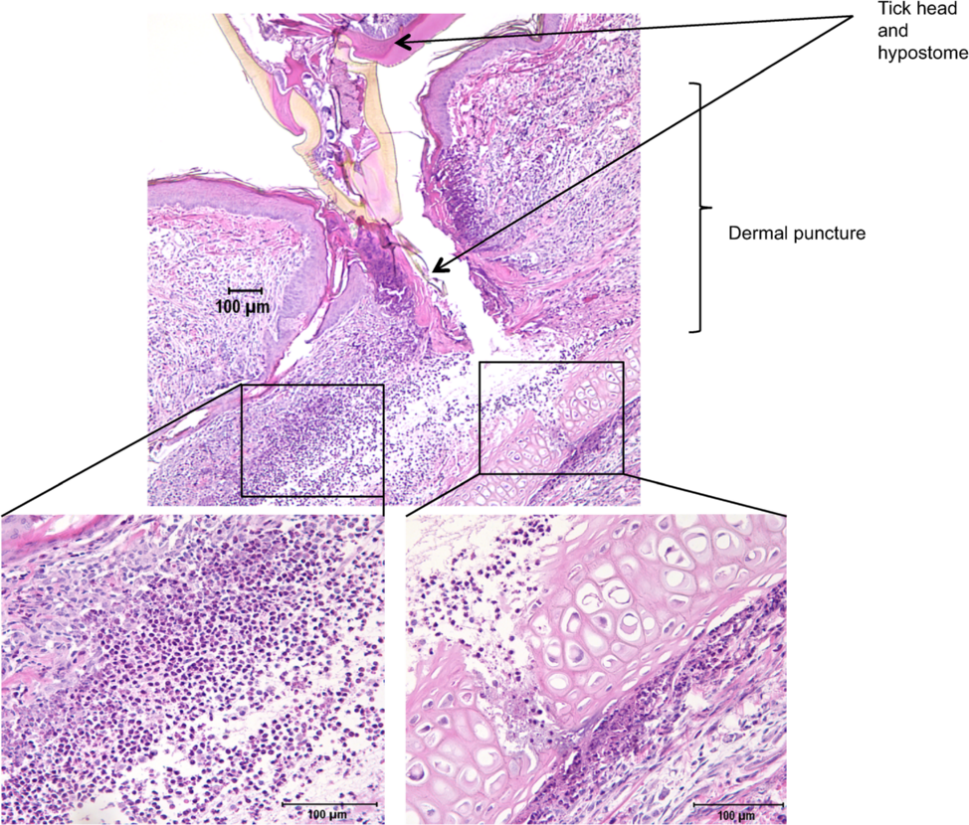
Tick feeding on rabbit ear. The tick hypostome is readily observed penetrating the epidermis (primary image) forming a linear tract of necrosis that extends through the superficial dermis and partially fractures the auricular cartilage (bottom right inset). Prominent histiocytic and granulocytic inflammation interpreted to represent a combination of heterophils and eosinophils (bottom left inset) surrounds the insertion of the tick hypostome within the dermis

While tick saliva is known to suppress multiple mediators of immunity, as outlined in the Background section, it does not shut down inflammation at the site of the tick bite. We obtained a cross-section of an adult *Ixodes scapularis* tick feeding on a rabbit and stained it with H&E. Rabbits are known to generate anti-tick immunity following repeated re-infestation [32]. Here, upon primary infestation, recruitment of inflammatory cell types by tick saliva is demonstrated. As shown in Fig. 1, the dermal puncture sight is surrounded by inflammatory cells. The infiltration of cells includes a significant population of histiocytes (derived from monocytes), indicating that the monocyte/macrophage is a relevant cell population for analysis of the impact of tick saliva on the response to *B. burgdorferi*. Also present is a population of granulocytic cells, as previously reported [32]. Thus, pro-inflammatory mediators (e.g. IL-8/CXCL8) that may be induced by exposure of skin fibroblasts to tick saliva should play a role in this response at the site of tick feeding.

### Effect of tick saliva on inflammatory mediator production by THP-1 cells as measured by ELISA

**Fig. 2:**
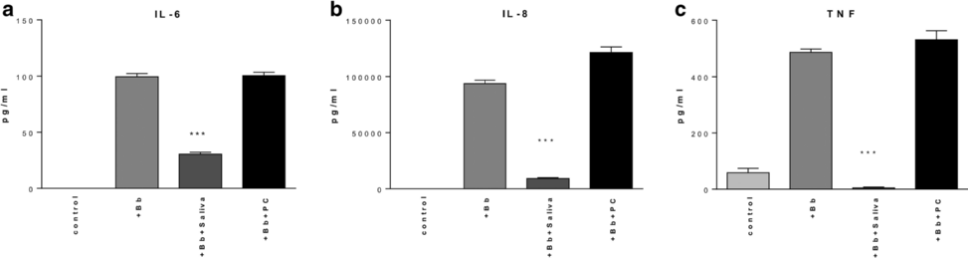
Effect of tick saliva on inflammatory mediator production by THP-1 cells, as measured by antigen-capture ELISA. Supernatants from THP-1 cells grown for 24 h: unstimulated control (control), incubated with *B. burgdorferi* (MOI 10:1) (+Bb) alone, *B. burgdorferi* and 64 μg saliva (+Bb + Saliva) or *B. burgdorferi* and 0.7 μM PC (+Bb + PC). Supernatants were subjected to antigen capture ELISA for IL-6 **a**, IL-8 **b** and TNF-α **c**. Cells exposed to *B. burgdorferi* plus tick saliva showed a significant decrease in inflammatory mediator production. ***P < 0.001

The inflammatory response mediated by nuclear factor-kB pathway (NF-kB) is thought to be a final common pathway for the translation of a variety of environmental insults, including those caused by blood-feeding arthropods, into inflammation [33]. To evaluate the expression of the pro-inflammatory mediators IL-6, IL-8 (CXCL8) and TNF-α by THP-1 cells stimulated with *B. burgdorferi*, supernatants were harvested at 12 and 24 h of incubation for analysis via antigen-capture ELISA. THP-1 cells were co-cultured with *B. burgdorferi* (MOI 10:1) either in the presence or absence of tick saliva (64 μg/ml protein). Prior experiments demonstrated that saliva had no effect on the production of these mediators by THP-1 cells (Additional file 1: Figure S1). At the 24-h (Fig. 2a–c) and 12-h time points (not shown), production of all three pro-inflammatory mediators was significantly inhibited (at least 50 %, *P* < 0.001) by treatment with saliva. PC added in the concentration range comparable to that found in the saliva pool used in this study induced no inhibition of inflammatory mediator production (Fig. 2a–c). Saliva alone did not elicit inflammatory mediator production (data not shown).

### Effect of tick saliva on inflammatory mediator production by THP-1 cells as measured by cytokine bead array

**Fig. 3:**
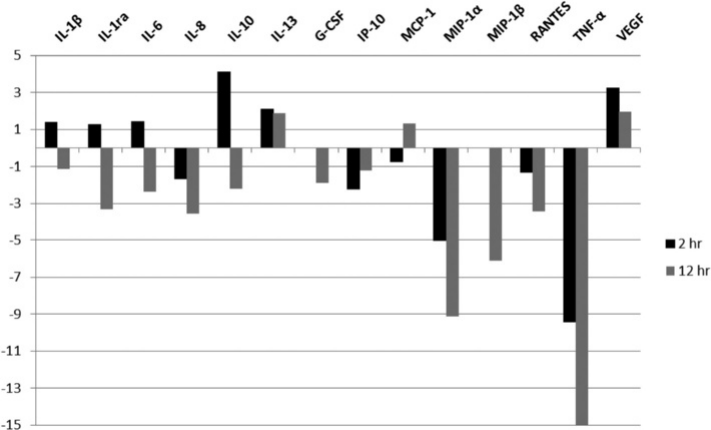
THP-1 response to tick saliva in the context of *B. burgdorferi* as measured via cytokine bead array. THP-1 supernatants collected after 2 and 12 h of stimulation with *B. burgdorferi* alone or in addition to saliva. The zero (baseline) is the level of stimulation with Bb only and the fold-change indicates that imparted by the addition of saliva. Supernatant analyte levels were measured in ng/ml for each time point and compared to determine the effect saliva had on the response of monocytes to *B. burgdorferi*

**Fig. 4:**
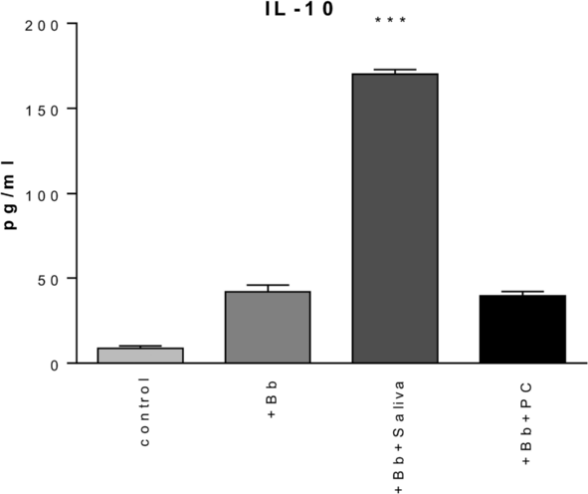
Effect of tick saliva on IL-10 production by THP-1 cells, as measured by 27-plex cytokine bead array. Supernatants obtained from THP-1 cells after 2-h incubations in medium alone (control), in the presence of *B. burgdorferi*, MOI 10:1 (+Bb), *B. burgdorferi* and 64 μg tick saliva, (+Bb + Saliva), or *B. burgdorferi* and 0.7 μM PC, (+Bb + PC), were subjected to cytokine bead array. Cells exposed to *B. burgdorferi* plus tick saliva showed a significant increase in IL-10 production. ***P < 0.001

The ELISA results showed that tick saliva is able to suppress inflammatory mediator production by THP-1 cells. In order to determine how widespread this effect was, multiplex cytokine bead arrays were used. THP-1 supernatants were collected after 2 and 12 h of stimulation with *B. burgdorferi* alone or in addition to either saliva or PC. Culture supernatants of THP-1 cells incubated in medium alone served as the negative control. Supernatant cytokine (analyte) levels were measured for each time point and compared with controls to determine the effect saliva had on the response of monocytes to *B. burgdorferi* (Table 1, Fig. 3). In some instances, the measured analyte was unaffected by saliva treatment (concentrations were comparable to those measured for THP-1 + *B. burgdorferi* only) and so these were recorded as “no effect.” As with the antigen-capture ELISA experiment, the production of IL-6, IL-8 (CXCL8) and TNF-α elicited by *B. burgdorferi* was significantly inhibited by tick saliva at the 12-h time point, as determined by the cytokine bead array (Table 1). In addition, the production of IL-1β, IL-1RA, IL-10, G-CSF, IP-10, MIP-1α, MIP-1β/CCL4, and RANTES also was inhibited after 12 h of incubation. In contrast, three analytes were enhanced by tick saliva at this time, namely, IL-13, MCP-1, and VEGF. The effect of tick saliva measured at 2-h post-stimulation with *B. burgdorferi* was markedly disparate from that at 12 h. The production of IL-1β, IL-6, IL-10, IL-12 (p70), IL-13, and VEGF was significantly enhanced. Indeed, the amount of IL-10 produced almost tripled by treatment with saliva when compared to that produced by THP-1 cells in the presence of *B. burgdorferi* alone (Fig. 4). At 2 h post-incubation, the following mediators were significantly inhibited: IL-8 (CXCL8), IP-10, MCP-1, MIP-1α, and RANTES, and all but MCP-1 remained inhibited after 12 h.

**Table 1:** Effect of tick saliva on inflammatory mediator (mean ± standard error) production by THP-1 cells in the presence of *B. burgdorferi* as measured by cytokine bead array

27-plex assay	2 h		12 h	
Mediator	Bb	Bb + saliva	Bb	Bb + saliva
IL-1β	2,793.9 ± 101.8	3,802.3 ± 430.3^a^	1662.6 ± 101.1	1,440.8 ± 12.7^a^
IL-1ra	143.2 ± 1.3	197.5 ± 11.0^a^	5481.2 ± 673.3	1,655.5 ± 28.6^b^
IL-6	162.8 ± 5.9	193.4 ± 21.3	299.9 ± 11.4	129.2 ± 4.3^b^
IL-8	42,586.9 ± 235.5	24,111.6 ± 482.3^b^	170,374.1 ± 12,978.4	48,064.5 ± 1,451.6^b^
IL-10	72.0 ± 6.8	296.1 ± 83.8^b^	553.0 ± 24.9	251.6 ± 9.7^b^
IL-13	94.6 ± 56.7	202.9 ± 74.0	289.1 ± 60.2	541.4 ± 82.4^a^
G-CSF	109.5 ± 9.3	63.1 ± 2.4^a^	675.1 ± 37.3	360.1 ± 13.3^b^
IP-10	1,392.6 ± 94.8	630.2 ± 48.8^b^	4,983.9 ± 1,283.1	4,084.2 ± 65.5
MCP-1	522.1 ± 19.0	389.1 ± 23.9^a^	5,190.9 ± 225.5	6,943.4 ± 295.6^a^
MIP-1α	21,311.5 ± 577.8	4,242.5 ± 300.1^b^	8,825.5 ± 550.8	967.2 ± 53.7^b^
RANTES	5,224.3 ± 164.1	3,934.6 ± 129.4^a^	19,835.9 ± 521.2	5774.2 ± 93.5^b^
TNF-α	54,937.7 ± 3,339.3	5,820.6 ± 232.5^b^	2,208.1 ± 190.0	66.9 ± 11.7^b^
VEGF	1,470.3 ± 270.0	4,772.8 ± 428.4^b^	19,852.6 ± 2,237.0	38,831.1 ± 243.0^b^

^a^Statistically significant compared to Bb only, as determined by two-tailed *t*-test, *P* < 0.05

^b^Statistically significant compared to Bb only, as determined by two-tailed * t*-test, *P* < 0.001

Importantly, the ability of tick salivary components to bind cytokines [34], chemokines [35] and growth factors [36] could obscure results, especially when the detection relies only on antigen-capture ELISA. We have therefore examined the impact of saliva using cytokine bead array, microarray and examined key pathways using RT-PCR.

### Effect of tick saliva on inflammatory mediator production by human and rhesus dermal fibroblasts

THP-1 cells may be considered as a model for circulating monocytes; these are often found in the dermis. Results from experiments with THP-1 cells indicated that tick saliva might have an inhibitory effect on the production of some pro-inflammatory mediators in skin macrophages, as well as enhance both pro- and anti-inflammatory mediator production in a time-dependent fashion. To evaluate whether these results could be extended to dermal cells of a lineage other than monocytic, we chose to examine primary dermal fibroblasts. Fibroblasts are capable of producing pro-inflammatory mediators, as well as extracellular matrix proteins. They are ubiquitous in the connective tissue of the dermis.

**Fig. 5:**
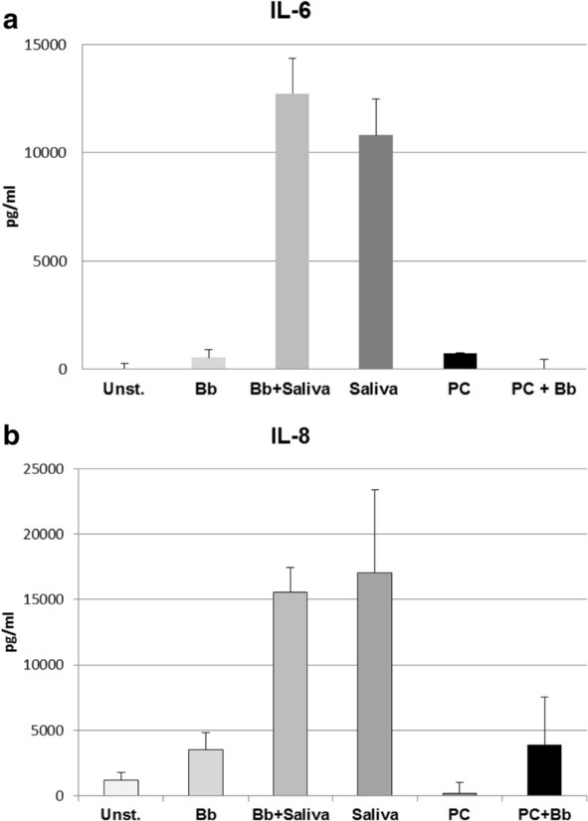
Effect of tick saliva on inflammatory mediator production by human dermal fibroblasts. Human fibroblasts were plated at 5 × 10^5^ cells per well and cultured for 24 h in the presence of medium alone (unst), with added live *B. burgdorferi* 10:1 (Bb), *B. burgdorferi* + 64 μg saliva (Bb + Saliva), saliva alone (Saliva), 5 mM pilocarpine control (PC), or Bb + PC. The concentrations of IL-6 **a** and IL-8 **b** were determined by antigen capture ELISA. Cells exposed to *B. burgdorferi* and tick saliva as well as cells exposed only to tick saliva showed a significant increase in inflammatory mediator production; **a** IL-6, *P* < 0.0001 and **b** IL-8, *P* < 0.0001. Each experiment was performed twice, in triplicate. Error bars represent standard deviation between three culture replicates tested in duplicate wells by ELISA

Human dermal fibroblasts were plated as described in Methods. Next, we examined how saliva affected pro-inflammatory mediator production when in the presence and absence of *B. burgdorferi*. Fibroblasts constitutively produced IL-6 when cultured in medium alone, and incubation with live *B. burgdorferi* (MOI 10:1) for 24 h resulted in increased production of this cytokine, as well as that of IL-8/CXCL8 (Fig. 5a, b). Surprisingly, production of both IL-6 and IL-8(CXCL8) by fibroblasts was significantly enhanced when tick saliva was added to the cultures (*t*-test: *t*_(5.54)_ = -17.909, *P* < 0.0001 for IL-6 and *t*_(8.66)_ = -160.5, *P* < 0.0001 for IL-8), even in the absence of *B. burgdorferi* (*t*-test: *t*_(5.50)_ = -14.64, *P* <0.0001 for IL-6 and *t*_(5.40)_ = -5.08, *P* = 0.0031 for IL-8; Fig. 5 a, b). To confirm that this stimulatory effect was not a unique attribute of the batch of tick saliva used in the fibroblast experiment, we re-tested saliva from this batch with THP-1 cells. As before, production of both IL-6 and IL-8(CXCL8) by THP-1 cells was enhanced by exposure to live spirochetes and significantly inhibited by saliva (not shown). The stimulatory effect of saliva on fibroblasts was not unique to human cells, as this phenomenon was also observed with rhesus macaque fibroblasts (not shown). Thus, saliva itself had a pro-inflammatory effect on dermal fibroblasts. This effect was not due to PC, as human fibroblasts incubated with PC at a concentration commensurate with that present in the saliva specimen had no significant effect on production of IL-6 or IL-8(CXCL8) by THP-1 cells.

### Effect of tick saliva on inflammatory mediator production by THP-1 cells as measured by DNA microarray

Results from ELISA and cytokine bead array experiments indicated that saliva has a widespread effect on pro- and anti-inflammatory mediator production by THP-1 cells in the presence of *B. burgdorferi*. To evaluate the extent of this effect on the expression of not only mediators but also receptors and pathways involved in inflammation, we conducted microarray experiments at two time points: 2 h and 12 h post-incubation of THP-1 cells with spirochetes alone, and with spirochetes plus tick saliva. In the analysis of affected genes, two broad categories of gene function dominated the response-immune function and apoptosis regulation. By restricting the analysis to genes significantly perturbed (1.5-fold or more) in the categories of immune regulation and apoptosis, we were able to characterize the impact of tick saliva on monocytic cells in the context of *B. burgdorferi* infection. (see Additional file 2: Table S1 and Tables 2 and 3) include lists of all immune function and apoptosis regulatory genes whose expression was affected by *B. burgdorferi* and *B. burgdorferi* plus tick saliva.

**Table 2:** Genes related to TLR2 signaling whose expression in THP-1 monocytes was affected by incubation with *B. burgdorferi* and *B. burgdorferi* plus tick saliva at 2 and 12 h post-incubation

Gene identifier	*B. burgdorferi*		*B. burgdorferi* + saliva	
	2 h fold-change	12 h	2 h fold-change	12 h
CD14	-1.2	na	na	na
ELK1	na^a^	-1	na	na
FOS	na	-1.6	1.7	na
IKBKG	na	4	na	na
IRAK1	2.6	-2.2	na	na
IRAK2	na	1.1	na	na
JUN	na	4.9	na	na
MAP2K3	-1.3	-2.1	na	na
MAP3K7	na	5.0	na	na
MAP3K7I	-2	na	na	na
MAP4K4	-3.3	4.9	1.3	na
MAPK8	-1.5	na	na	na
MAPK12	-1.1	na	na	na
MAPK13	-1.1	na	na	na
MAPK14	-4.2	3.6	na	1.3
MYD88	na	1.5	na	na
TIRAP	-1.0	4.1	na	na
TLR1	na	2.6	na	na
TLR2	na	na	-1.7	na
TLR7	na	-4.9	na	na
TLR8	na	1.6	na	na
TLR9	na	3.2	na	na
TOLLIP	na	5.4	na	na

^a^na, no data were generated for this gene identifier at this particular time point under those experimental conditions

**Table 3:** Microarray analysis of genes related to the IL-10 pathway whose expression in THP-1 monocytes was affected by incubation with *B. burgdorferi* and tick saliva at 2 and 12 h post-incubation

Gene identifier	*B. burgdorferi*		*B. burgdorferi* + saliva	
	2 h	12 h fold-change	2 h	12 h
BCL3	2.2	na	1.3	na
BLVRE	na^a^	-2.6	na	na
CCR1	na	na	-1.1	na
CD14	-1.2	na	na	na
ELK1	na	-1.0	na	na
FCGR2A	na	-1.9	na	na
FCGR2B	na	-1.3	na	na
FCGR2C	na	-1.7	na	na
FOS	na	-1.6	na	na
HMOX1	na	2.0	na	na
IKBKG	na	4.0	na	na
IL10RA	-1.8	na	na	na
IL10RB	na	-1.1	na	na
IL1B	3.3	2.7	na	na
IL1F5	-2.3	na	na	na
IL1F9	na	5.1	na	na
IL1F10	6.7	na	na	na
IL1R1	na	na	na	-1.3
JUN	na	4.9	na	na
MAP2K3	-1.3	-2.1	na	na
MAP3K7	na	5.0	na	na
MAP3KK7I	-2.0	na	na	na
MAP4K4	-3.2	4.9	1.3	na
MAPK8	-1.5	na	na	na
MAPK12	-1.1	-2.9	na	na
MAPK13	-1.1	na	na	na
MAPK14	-4.2	3.6	na	1.3
SOCS3	na	5.8	1.4	na
STAT3	na	-1.0	na	na
SP1	-1.6	na	na	na
TNF-α	na	4.5	na	na
TYK2	na	-1.9	na	na

^a^na, no data were generated for this gene identifier at this particular time point under those experimental conditions

In particular, apoptosis facilitators, such as the BCL-2 like genes were upregulated by *B. burgdorferi*, but down-regulated by tick saliva (Additional file 2: Table S1c, d and Additional file 3: Table S2c, d). The immune -related genes dominated the responses by monocytic cells to both *B. burgdorferi* and *B. burgdorferi* with saliva (Additional file 2: Table. S1a, b and Additional file 3: Table S2a, b).

### 2-h time point

Treatment with *B. burgdorferi* alone for two hours induced down-regulation of 982 genes and up-regulation of 1015 genes. By 2 h post-incubation, genes that were solely up-regulated by *B. burgdorferi* include the pro-inflammatory mediators TNF-α, IL-6, and IL-8(CXCL8). *Borrelia burgdorferi* also induced up-regulation of the chemokines MIP-1α/CCL3 and RANTES/CCL5, both of which attract immune cells to sites of inflammation (Table 3 and Additional file 2, Table S1a, b). TLR2 was not identified as being up-regulated at this particular time point; however, additional experiments in our laboratory have shown that its expression is up-regulated by as early as 30 min post-incubation with *B. burgdorferi*. Specifically, TLR2 was up-regulated 1.6 fold at 30 min by THP-1 cells following co-incubation with *B. burgdorferi* (data not shown). Due to the necessity of TLR2 for the induction of inflammation by *B. burgdorferi* [37], and the multiple TLR pathway genes (MYD88, IRAKs, IkBKG, etc.) affected, we elected to examine TLR2 expression closer, with qRT-PCR.

MAP4K4, which encodes a protein kinase and is a member of the serine/threonine protein kinase family was also down-regulated by treatment with *B. burgdorferi* alone by 2 h post-incubation. (Table 3). MAP4K4 has been shown to activate MAPK8/JNK. However, it has been suggested that this kinase may function through an alternative MAP kinase cascade and mediate the TNF-α signaling pathway. The addition of saliva caused a down-regulation of the expression of TLR2, TNF-α, MIP-1αCCL3, IL-17, and MIP-1β/CCL4 by 2 h post-incubation (Table 2 and Additional file 2, Table S1b). Tick saliva contains copious amounts of eicosanoids in the form of prostaglandins. Prostaglandins have both pro-inflammatory functions, including vasodilatory and fever-inducing, as well as immune modulatory functions such as inhibiting TNF-α, IL-1β and IL-12. Prostaglandins can also stimulate the production of IL-10 and therefore indirectly inhibit many aspects of cell-mediated immunity [38].

By 2 h post-incubation with *B. burgdorferi* and saliva, a total of only 803 genes were down-regulated, while 1103 genes were up-regulated. At this early time point, treatment with saliva caused an up-regulation of the expression of SOCS3, as well as MAP4K4. Conversely, saliva treatment at this time point had an inhibitory effect on the expression of CCR1, a receptor for several pro-inflammatory chemokines, including MIP-1α, and RANTES.

### 12-h time point

A total of 7918 genes were down-regulated and 3866 genes were up-regulated after 12 h of incubation of THP-1 monocytes with *B. burgdorferi* alone. The addition of saliva and *B. burgdorferi* for the same time period caused the up-regulation of 1203 genes and the down-regulation of 1371 genes. By 12 h post-incubation, among the genes that were affected by treatment with *B. burgdorferi* alone were the pro-inflammatory mediators IL-1β and TNF-α, which were up-regulated. By this time point, the expression of MAP4K4, a potential mediator of TNF-α signaling, was also up-regulated by nearly 5-fold. Down-regulated genes from this sample included TLR-7, MHC genes, complement-related genes and IL-17 receptors (Additional file 3, Table S2b). Some of the genes up-regulated in the saliva-treated samples included TNF-α and IL10RA (Table 2, Additional file 3, Table S2a). Down-regulated genes in the same sample included IL-13, IL-19, and TGFβ.

Those genes affected by treatment with both spirochetes alone, and with treatment including spirochetes plus tick saliva are shown in Tables 2 and 3, and Additional files 2 and 3. With respect to genes affected by *B. burgdorferi*, and also modulated by tick saliva (Tables 4, 5, 6 and 7), several genes involved in the innate response, especially at 2 h are down-regulated (Table 4). Most notably, CCL4 (also known as macrophage inflammatory protein 1β) and CCL4L2 are significantly down-regulated by saliva. This may help shut down recruitment of macrophages and NK cells to the site of tick feeding. The down-regulation of TGFβ and HLA may also serve to reduce inflammation and dampen the onset of adaptive immunity. Genes that promote apoptosis (AIFM1, BCL2L14, CARD15) appear also to be down-regulated by tick saliva at 2 and 12 h (Table 4, 5 and 6); however, the pro-apoptotic gene BAX is up-regulated at 12 h (Table 7). Appropriately, cell signaling molecules such as the mitogen-activated protein kinases and NFkB genes are also perturbed with *B. burgdorferi* and tick saliva.

**Table 4:** *B. burgdorferi*-perturbed genes down-regulated after 2 h of stimulation

Gene	Relative expression	Description
MMP23B	-0.1740859	Matrix metallopeptidase 23A (pseudogene); matrix metallopeptidase 23B
CCL4	-5.833905	Chemokine (C-C motif) ligand 4
CD209	-0.6862013	CD209 molecule
AIFM1	-0.4024457	Apoptosis-inducing factor, mitochondrion-associated, 1
KIR3DL1	-0.6602146	Killer cell immunoglobulin-like receptor
ITIH5	-0.2748285	Inter-alpha (globulin) inhibitor H5
IL6R	-0.0037182	Interleukin 6 receptor
MAPK8IP1	-0.2555757	Mitogen-activated protein kinase 8 interacting protein 1
MAPK8IP2	-0.361055	Mitogen-activated protein kinase 8 interacting protein 2
CCL4L2	-3.7781608	Chemokine (C-C motif) ligand 4-like 1; chemokine (C-C motif) ligand 4-like 2
MAP4K4	-0.151645	Mitogen-activated protein kinase kinase kinase kinase 4
GPR56	-0.0379491	G protein-coupled receptor 56
BCL2L14	-1.0708373	BCL2-like 14 (apoptosis facilitator)
LTBP4	-0.3332754	Latent transforming growth factor beta binding protein 4
TRAF4	-0.4525453	TNF receptor-associated factor 4
IL17RC	-0.4447441	Interleukin 17 receptor C

**Fig. 6:**
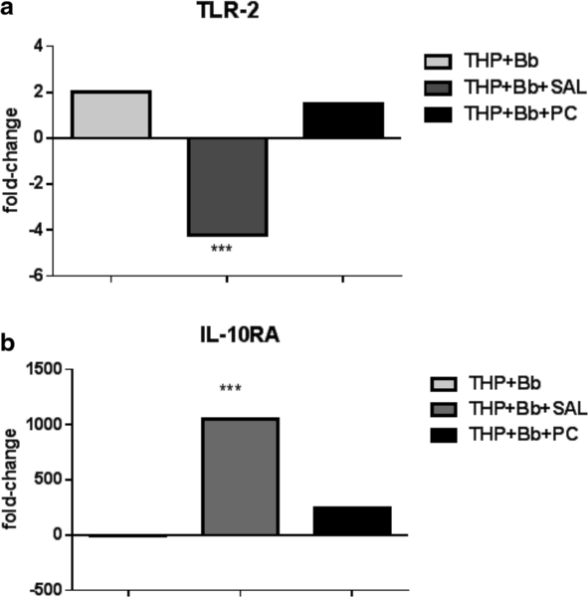
TLR2 and IL-10RA qRT-PCR results THP-1 RNA was extracted from 2-h cultures stimulated with *B. burgdorferi* (1:10) alone, *B. burgdorferi* and saliva or *B. burgdorferi* and 0.7 μM PC. Real-time PCR was performed for TLR2 and IL-10RA relative expression levels. **a**, Transcript levels from cells exposed to *B. burgdorferi* and saliva showed a significant decrease in TLR2 expression at 2 h post-stimulation (***P = 0.001); **b**, Transcript levels from cells exposed to *B. burgdorferi* and saliva showed a significant increase in IL-10RA expression (***P = 0.015)

**Table 5:** *B. burgdorferi*-perturbed genes up-regulated after 2 h of stimulation

Gene	Relative expression	Description
IGHG1	0.07896333	Variable 3–11 (gene/pseudogene); immunoglobulin heavy variable 4–31; immunoglobulin heavy locus
HIF1AN	1.40558447	Hypoxia inducible factor 1, alpha subunit inhibitor
FGFR3	3.37091511	Fibroblast growth factor receptor 3
CD6	2.9080693	CD6 molecule
CD300E	0.65831278	CD300e molecule
CD200	2.10422558	CD200 molecule
GPR89A	1.7942643	G protein-coupled receptor
BAT3	0.09701591	HLA-B associated transcript 3
IL15RA	0.28488171	Interleukin 15 receptor, alpha
NCF1	0.41282425	Neutrophil cytosolic factor 1
MAP3K7IP1	0.13999618	Mitogen-activated protein kinase kinase kinase 7 interacting protein 1
RAB1A	0.68755658	RAB1A, member RAS oncogene family
IL12RB1	0.78907124	Interleukin 12 receptor, beta 1
TNFRSF13B	0.02283238	Tumor necrosis factor receptor superfamily, member 13B
IL1F10	1.59882878	Interleukin 1 family, member 10 (theta)
BAD	0.44343992	BCL2-associated agonist of cell death
RASSF7	1.95038068	Ras association (RalGDS/AF-6) domain family (N-terminal) member 7
RASGRF1	0.28493451	Ras protein-specific guanine nucleotide-releasing factor 1
CNOT7	0.31888622	CCR4-NOT transcription complex, subunit 7
LTBP1	2.24423737	Latent transforming growth factor beta binding protein 1
CD44	0.61183676	CD44 molecule (Indian blood group)

**Table 6:** *B. burgdorferi*-perturbed genes down-regulated after 12 h of stimulation

Gene	Relative expression	Description
BCL7B	-0.00593926	B-cell CLL/lymphoma 7B
CARD15	-2.990009101	Caspase recruitment domain family, member 15
HLA-DMB	-7.115679019	Major histocompatibility complex, class II, DM beta
RAB4B	-6.491	RAB4B, member RAS oncogene family
RASSF7	-4.73653	Ras association (RalGDS/AF-6) domain family (N-terminal) member 7
TGFB1	-4.967537664	Transforming growth factor, beta 1

**Table 7:** *B. burgdorferi*-perturbed genes up-regulated after 12 h of stimulation

Gene	Relative expression	Description
BAX	3.864113319	BCL2-associated X protein
CD79B	4.848595465	CD79b molecule, immunoglobulin-associated beta
MMP25	3.984962116	Matrix metallopeptidase 25
NFKB2	1.78061484	Nuclear factor of kappa light polypeptide gene enhancer in B-cells 2 (p49/p100)
NFKBIL1	2.477201984	Nuclear factor of kappa light polypeptide gene enhancer in B-cells inhibitor-like 1
RASGRF1	2.626291514	Ras protein-specific guanine nucleotide-releasing factor 1

### Real-time reverse-transcriptase PCR

To verify the results of the microarray experiments, we conducted quantitative real-time reverse transcriptase PCR on two genes of interest, *TLR2* and *IL-10RA* (Fig. 6 a, b). RNA harvested after 2 h of incubation was used as template. *TLR2* expression was significantly diminished in those samples obtained from *B. burgdorferi* plus saliva treatment when compared to RNA obtained from samples not receiving any saliva treatment. RNA obtained after 12 h of incubation was used for the qRT-PCR analysis of *IL-10RA* expression. Relative expression of *IL-10RA* was significantly elevated in RNA samples obtained from the *B. burgdorferi* + saliva treatment compared to no saliva treatment. These data confirm the validity of microarray data.

## Discussion

The goal of this study was to examine the effect of tick saliva on monocytes and primary dermal fibroblasts when these cells were exposed to *B. burgdorferi*. These two cell types are both found in the skin, where the first point of contact between the vector, pathogen, and host is located. In these studies, we utilized a human cell line (THP-1) for the monocyte model, and primary cells for the fibroblast model. While a pure population of human primary monocyte/macrophages may have yielded more relevant findings, our cytokine results are in agreement with published data [39] and expand upon available information regarding gene expression. The surface of the skin is constantly exposed to potential pathogens, yet wide-ranging skin infections are relatively rare, owing to efficacy of the immune response by the skin [40]. Supporting this notion is the demonstration that dermal fibroblasts are capable of secreting several immunomodulators, including IFN-γ, PGE_2,_ IL-1, IL-6, and IL-8 (CXCL8) while monocytes and macrophages present in the skin play roles in immune defense, inflammation and tissue remodelling [41]. Along with monocyte/macrophages, Langerhans cells and dermal dendritic cells (DCs) serve as the antigen-presenting cells (APC) which act as key regulators bridging the innate and adaptive responses. These cells pick up antigen [42] and migrate to the regional lymph nodes, where they present antigen to effector T cells. Among the demonstrated effects of *Ixodes* tick saliva on dendritic cells (reviewed in [43]) are inhibition of phagocytosis [44], suppression of inflammatory signals [44, 45] and interference with antigen presentation [46, 47].

In addition, recent studies have shown that co-incubation of keratinocytes and dermal fibroblasts with *B. burgdorferi* induce an inflammatory response [48, 49]. Mediators such as IL-8 (CXCL8) and the antimicrobial peptides human β-defensin-2 and cathelicidin LL-37 were induced upon co-incubation with *B. burgdorferi* by both keratinocytes and fibroblasts [48, 49]. Our results are in agreement with these studies, but are expanded to include the effect of saliva. In a subsequent study, Marchal et al. [4] showed that tick salivary gland extract has an inhibitory effect on the expression of inflammatory mediators in keratinocytes when co-incubated with *B. burgdorferi.* These results support the current paradigm that molecules present in tick saliva have the capacity to inhibit many host immune functions [7]. As regulators of inflammation, monocytes are able to produce pro-inflammatory mediators such as IL-8 (CXCL8), a chemokine that controls the influx of inflammatory and immune cells to sites of injury and infection, and IL-6, a key B cell differentiation factor.

A fundamental element in the host’s innate immune response is inflammation. Inflammation causes increased vascular permeability, which promotes the migration and influx of immune cells to areas of tissue damage. The current paradigm, widely described in the literature, suggests that, in order to maintain feeding, hard ticks require anti-inflammatory mediators in their saliva so that they may feed to repletion and remain relatively undetected by their host. In addition to several pharmacologically active compounds, saliva does contain anti-inflammatory agents. Hard ticks must remain attached to their host for several days without initiating a wound healing response at the site of dermal tissue damage. Our initial goal was to test the hypothesis that tick saliva would have global suppressive or inhibitory effects on the production/transcription of pro-inflammatory mediators secreted by dermal cells such as blood monocytes and fibroblasts. However, our results suggest that the current paradigm may be an oversimplification of a more complex interplay between host, vector and pathogen. Tick saliva was able not only to inhibit but also enhance inflammatory mediator production and transcription elicited by *B. burgdorferi* in monocytes. In fibroblasts, it was able to elicit production of pro-inflammatory mediators even in the absence of the spirochete. While a previous study indicated that tick saliva can have a cytotoxic effect on fibroblasts [49], or slow cell proliferation [36] we did not observe decreased viability and our study was too short to measure the impact on proliferation. The discrepancy in cytotoxicity could be due to differences in methodology, with our use of saliva versus salivary gland extract. Moreover, saliva effects on monocytes in some cases depended on the length of exposure to the saliva, and could switch from early inhibitory to late enhancing, and vice versa.

If we inspect the area of tick feeding histologically (Fig. 1), the dermal puncture site is not free of inflammatory cells. Indeed, inflammatory cells are recruited to the abrasion site of the dermis and even below the basement membrane. The wound-healing response is not globally down-regulated by tick saliva, but is limited to the dermis. Relatively little inflammation is seen in the epidermis at the site of feeding.

It has previously been demonstrated that salivary gland extracts (SGE) from *I. ricinus* inhibits production of IFN-γ through the up-regulation of IL-10 [50]. Furthermore, studies utilizing C3H/HeN mice demonstrated the effects of infestation with *I. scapularis* nymphs on chemokine production. Repeated infestations with pathogen-free *I. scapularis* nymphs decreased the production of macrophage-derived chemokines such as IL-1β, IL-6 and TNF-α and increased the production of T lymphocyte cytokines such as IL-2, IL-4, and IL-10 in C3H/HeN mice [51]. Our experiments with *I. scapularis* saliva, live *B. burgdorferi*, and THP-1 monocytes (ELISAs, multiplex cytokine bead array, microarray and qRT-PCR) yielded data that support these earlier findings as well as our initial hypothesis. Certain components in saliva suppress or inhibit the expression of pro-inflammatory mediators by these monocytes. THP-1 monocytes treated with tick saliva in the presence of live *B. burgdorferi* demonstrate that saliva inhibits several pro-inflammatory mediators, including IL-6, IL-8 (CXCL8), and TNF-α. Furthermore, treatment with saliva enhances the expression of several anti-inflammatory mediators by THP-1 monocytes, including, IL-10, IL-10RA, and IL-13, which can down-regulate macrophage activity, and inhibit the production of pro-inflammatory cytokines and chemokines. It has also been previously demonstrated that interleukin-10 (IL-10), produced by THP-1 monocytes in response to *B. burgdorferi*, inhibits the production of concomitantly elicited inflammatory cytokines [52]. Thus, IL-10 could potentially down-regulate inflammatory and microbicidal effector mechanisms of the innate immune response to a *B. burgdorferi* infection, facilitating the establishment of the spirochete. Also consistent with the effect of *B. burgdorferi* [53] and tick saliva [54] on vascular permeability is the upregulation of VEGF production by both, as shown in Table 1.

TLR2 is a key receptor involved in recognition of *B. burgdorferi* by monocytes/macrophages. Our THP-1 monocyte microarray results demonstrate that tick saliva in the presence of *B. burgdorferi* suppresses TLR2 expression in monocytes by as early as 2 h post-stimulation (Table 2 and Additional file 2, Table S1b). This may represent one of the earliest points of interference by the tick on the host’s innate immune response. Similar results have also been obtained using dendritic cells [55]. TLR2 inhibition has downstream effects on the ability of monocytes to produce several pro-inflammatory mediators. PAMP recognition by TLRs initiates various innate immune responses including phagocytosis, production of antimicrobial compounds and inflammatory mediators, which in turn induce the killing of microorganisms and initiate the acquired immune response. Microbial infection such as that caused by *B. burgdorferi* involving skin damage through injury (tick feeding) leads to the activation of the inflammatory response by activation of several cellular signalling pathways, including the NF-kB via TLR2. NF-KB-mediated gene transcription can induce the expression of primary cytokine and chemokine production in monocytes/macrophages, fibroblasts, and other resident skin cells [56].

Addition of *B. burgdorferi* perturbed several genes, including SOCS3, SOCS4 and SOCS5, which were up-regulated (see Additional file 2, Table S1a; Additional file 3: Table S2a) and play a role in suppressing cytokine and chemokine signalling by binding to and inhibiting JAK2 kinase. When saliva was added, SOCS3 remained upregulated (1.28-fold) at the 2-h time point (data not shown), thus indicating that saliva does not curb this mode of immunosuppression. SOCS3 is involved in negative regulation of cytokines that signal through the JAK/STAT pathway [57], whereas SOCS4 and 5 appear to regulate the epidermal growth factor receptor [58], also seen down-regulated at 12 h. By 12 h post-incubation with *B. burgdorferi* and saliva, THP-1 expression of the heterodimeric receptor for IL-10, IL10RA, was increased by over 2-fold. This upregulation is significant because of the role that IL10RA plays in IL-10-Stat3 signalling. IL-10 binds to IL-10RA which then activates Jak1 and Tyk2 followed by Stat3 phosphorylation [59]. Stat3 is at least partially required for the inhibitory functions of IL-10 and induces the expression of SOCS3 which regulates a variety of cytokine signalling pathways, including IL-6 [59]. Indeed, the expression of SOCS genes is induced by various cytokines, including IL-6, IL-10, and IFN-γ. The observation that *B. burgdorferi* stimulates an increase in the expression of SOCS3 is therefore not unexpected.

A prominent response was the downregulation of CCL4 and CCL4L2 (ligand) by tick saliva. The decrease in expression of CCL4 was nearly 6-fold (Table 4) from that induced by *B. burgdorferi* alone. MIP-1 proteins mediate their effects by binding to cell surface CC chemokine receptors, which belong to the G-protein-coupled receptor superfamily. The high affinity interactions of receptor binding induce a subsequent cascade of intracellular events that rapidly leads to a wide range of target cell functions including chemotaxis, degranulation, phagocytosis, and mediator synthesis [60]. This reduction in the amount of chemoattractive inflammatory mediator may thus be a key element to saliva-aided pathogen survival in the skin during transmission.

In summary, microarray and qRT-PCR experiments using THP-1 monocytes co-cultured with *B. burgdorferi* and saliva demonstrate inhibition of TLR2 expression. Furthermore, we showed that saliva stimulates IL-10 production by 2 h of co-incubation, and IL-10RA expression is up-regulated by 12 h of co-incubation. IL-10 both down-regulates expression of Th1 type cytokines, and blocks NF-kappa B activity, while IL-10RA mediates the immunosuppressive effects of IL-10.

Experiments with human and rhesus primary dermal fibroblasts yielded results which do not support our initial hypothesis. ELISAs conducted with supernatants obtained from fibroblasts co-cultured with *B. burgdorferi* and saliva demonstrated that saliva elicits pro-inflammatory mediator production by these cells. Saliva does not inhibit IL-6, nor does it inhibit IL-8 (CXCL8), but rather, it significantly enhances production of both mediators. These results demonstrate that saliva has the opposite effect on primary dermal fibroblasts compared to its effect on THP-1 monocytes. Even in the absence of pathogen, tick saliva stimulates the expression of certain pro-inflammatory mediators by these cells. Fibroblasts secrete pro-inflammatory mediators, yet inflammation may be averted due to the counter measures imparted by the tick’s saliva on cells of the innate immune system, typified by monocytes/macrophages. Furthermore, recent research has suggested that anti-inflammatory agents present in tick saliva are capable of suppressing the release of local growth factors such as PDGF, EGF and TGFα, which play critical roles in the recruitment of fibroblasts into the site of tissue damage [18]. Studies conducted by Kramer et al. [61] demonstrate that SGE from *Dermacentor variabilis* suppresses the ability of fibroblasts to repair injury. Additional experiments show that SGE and saliva can inhibit fibroblast migration and reduce ERK activity [61].

The issue of timing may be a factor in the prevention of inflammation. Tick saliva may stimulate the production of inflammatory mediators by fibroblasts at the site of feeding early on, recruiting monocytes/macrophages to the site. However, the inhibition of monocytic responses at this point may prevent further inflammation and recruitment and/or stimulation of the cells of the adaptive immune response from the regional lymph nodes.

## Conclusions

Our results indicate that saliva can both suppress and stimulate the expression of pro-inflammatory mediators such as IL-6 and IL-8(CXCL8), depending on the cell type, and suggest that fibroblasts may be involved in immune responses that the tick is ultimately able to evade. The main conclusions that may be drawn from this study include the following: (i) saliva, while able to inhibit key pro-inflammatory pathways, is not a universal inhibitor of inflammation; and (ii) the effects of saliva in this regard are crucially dependent on the length of interaction with host cells, and on the type of host cell involved.

## Abbreviations

ELISA*,* enzyme-linked immunosorbent assay; HLA, human leukocyte antigen; MOI, multiplicity of infection; PAMP, pathogen-associated molecular pattern; PC, pilocarpine; qRT-PCR*,* quantitative real-time PCR; TLR2, toll like receptor 2; TNF, Tumor necrosis factor.

## Additional files

Additional file 1: Figure S1. Comparison of THP-1 responses to *B. burgdorferi* and saliva. Levels of IL-6 (top panel) and IL-8 (bottom panel) were compared when THP-1 cells were incubated with *B. burgdorferi* and 2 different dilutions of saliva (1:20 and 1:500). (TIF 379 kb) Additional file 2: Table S1. Genes perturbed at 2 h post-stimulation. (DOCX 41 kb) Additional file 3: Table S2. Genes perturbed at 12 h post-stimulation. (DOCX 43 kb)
