# Supplementary figures and images for: Immunomodulatory effects of tick saliva on dermal cells exposed to Borrelia burgdorferi, the agent of Lyme disease
Source: Parasit Vectors. 2016 Jul 8;9:394. doi: 10.1186/s13071-016-1638-7 (PMC4938952; doi:10.1186/s13071-016-1638-7)

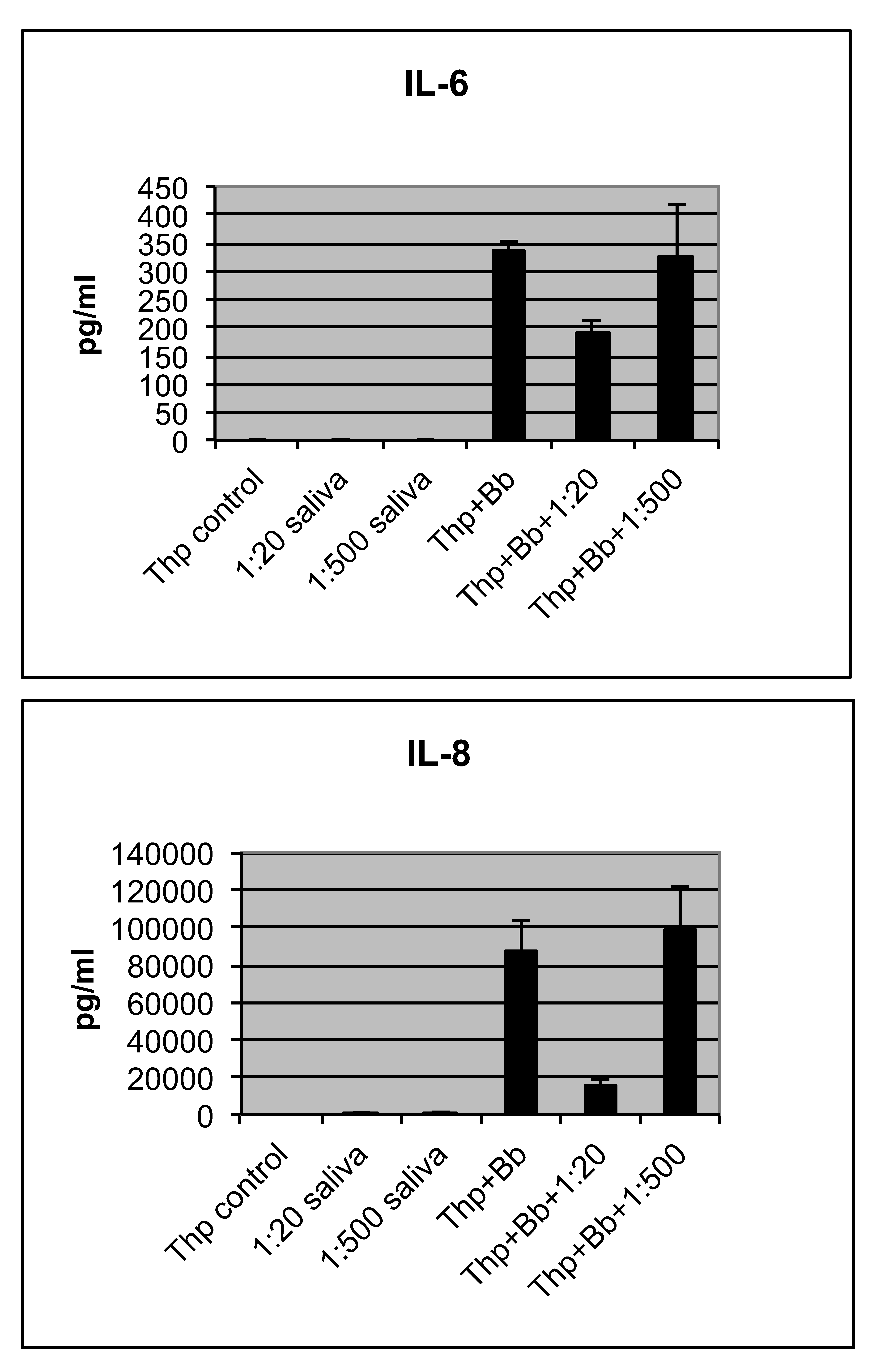

Supplement: Additional file 1: Figure S1. — Comparison of THP-1 responses to B. burgdorferi and saliva. Levels of IL-6 (top panel) and IL-8 (bottom panel) were compared when THP-1 cells were incubated with B. burgdorferi and 2 different dilutions of saliva (1:20 and 1:500). (TIF 379 kb) [file 13071_2016_1638_MOESM1_ESM.tif]
