# Supplementary material for: Immunomodulatory effects of tick saliva on dermal cells exposed to Borrelia burgdorferi, the agent of Lyme disease
Source: Parasit Vectors. 2016 Jul 8;9:394. doi: 10.1186/s13071-016-1638-7 (PMC4938952; doi:10.1186/s13071-016-1638-7)
Supplement: Additional file 2: Table S1. — Genes perturbed at 2 h post-stimulation. (DOCX 41 kb) [file 13071_2016_1638_MOESM2_ESM.docx]

| ***B. burgdorferi* only**  Supplementary Table 1a. Immune function genes upregulated at 2 hrs post-stimulation. | | | | | ***B. burgdorferi* + tick saliva** | | |
| --- | --- | --- | --- | --- | --- | --- | --- |
| *Gene name* | | *Fold regulation* | *Gene description* | | *Gene name* | *Fold regulation* | *Gene description* |
| \|  \| **Immune Function** \|  \| \| --- \| --- \| --- \| | | | | | | | |
| CCL22 | 8.775 | | | chemokine (C-C motif) ligand 22 | CSF3R | 1.571337 | colony stimulating factor 3 receptor (granulocyte) |
| CCL4 | 4.654 | | | chemokine (C-C motif) ligand 4 | TGFBI | 1.617561 | transforming growth factor, beta-induced, 68kDa |
| CCL4L2 | 2.208 | | | chemokine (C-C motif) ligand 4-like 1; chemokine (C-C motif) ligand 4-like 2 | CD93 | 1.622119 | CD93 molecule |
| CD300LB | 2.522 | | | CD300 molecule-like family member b | CCL14 | 1.660683 | chemokine (C-C motif) ligand 14; chemokine (C-C motif) ligand 15 |
| CD44 | 8.139 | | | CD44 molecule (Indian blood group) | CD47 | 1.663732 | CD47 molecule |
| CSF2RB | 4.301 | | | colony stimulating factor 2 receptor, beta, low-affinity (granulocyte-macrophage) | STAT1 | 1.709406 | signal transducer and activator of transcription 1, 91kDa |
| CXCL1 | 8.828 | | | chemokine (C-X-C motif) ligand 1 (melanoma growth stimulating activity, alpha) | CD74 | 1.860105 | CD74 molecule, major histocompatibility complex, class II invariant chain |
| CXCL2 | 6.781 | | | chemokine (C-X-C motif) ligand 2 | IL19 | 1.919571 | interleukin 19 |
| IFIT2 | 3.461 | | | interferon-induced protein with tetratricopeptide repeats 2 | IL16 | 1.952645 | interleukin 16 (lymphocyte chemoattractant factor) |
| IFNAR1 | 2.958 | | | interferon (alpha, beta and omega) receptor 1 | IGJ | 2.026558 | immunoglobulin J polypeptide, linker protein for immunoglobulin alpha and mu polypeptides |
| IFNAR2 | 6.087 | | | interferon (alpha, beta and omega) receptor 2 | CCR7 | 2.668967 | chemokine (C-C motif) receptor 7 |
| IGKV1D-13 | 3.018 | | | immunoglobulin kappa variable 1D-13 | TNFAIP8L3 | 2.704645 | tumor necrosis factor, alpha-induced protein 8-like 3 |
| IL17RE | 6.034 | | | interleukin 17 receptor E | IL7R | 2.974574 | interleukin 7 receptor |
| IL1B | 3.305 | | | interleukin 1, beta | IL24 | 3.020172 | interleukin 24 |
| IL1F10 | 5.059 | | | interleukin 1 family, member 10 (theta) | CXCR4 | 3.153714 | chemokine (C-X-C motif) receptor 4 |
| IL6ST | 3.638 | | | interleukin 6 signal transducer (gp130, oncostatin M receptor) | IGKV1D-13 | 4.289018 | immunoglobulin kappa variable 1D-13 |
| ILF3 | 7.139 | | | interleukin enhancer binding factor 3, 90kDa |  |  |  |
| IRAK1 | 2.569 | | | interleukin-1 receptor-associated kinase 1 |  |  |  |
| IRF1 | 5.362 | | | interferon regulatory factor 1 |  |  |  |
| LENG1 | 7.756 | | | leukocyte receptor cluster (LRC) member 1 |  |  |  |
|  |  | | |  |  |  |  |
| NFKBIL1 | 3.840 | | | nuclear factor of kappa light polypeptide gene enhancer in B-cells inhibitor-like 1 |  |  |  |
| NOS2A | 2.870 | | | nitric oxide synthase 2a, inducible |  |  |  |
|  |  | | |  |  |  |  |
| SOCS4 | 6.040 | | | suppressor of cytokine signaling 4 |  |  |  |
| TGFB3 | 6.931 | | | transforming growth factor, beta 3 |  |  |  |
| TNFRSF10C | 4.834 | | | tumor necrosis factor receptor superfamily, member 10c, decoy without an intracellular domain |  |  |  |
| TNFRSF6B | 2.708 | | | tumor necrosis factor receptor superfamily, member 6b, decoy; regulator of telomere elongation helicase 1 |  |  |  |
| TNFRSF9 | 6.813 | | | tumor necrosis factor receptor superfamily, member 4 |  |  |  |
| TNIP3 | 7.531 | | | TNFAIP3 interacting protein 3 |  |  |  |
| TRAF3IP2  Supplementary Table 1b. Immune function genes downregulated at 2 hrs post-stimulation. | 8.111 | | | TRAF3 interacting protein 2 |  |  |  |

| ***B. burgdorferi* only (2 hrs)-downregulated** | | | ***B. burgdorferi* + tick saliva (2 hrs)-downregulated** | | |
| --- | --- | --- | --- | --- | --- |
| *Gene name* | *Fold regulation* | *Gene description* | *Gene name* | *Fold regulation* | *Gene description* |
| \| **Immune Function** \| \| --- \| | | | | | |
|  |  |  |  |  |  |
| C1QC | -2.1206 | complement component 1, q subcomponent, C chain | IGSF4B | -2.34648 | immunoglobulin superfamily, member 4B |
| C1S | -1.50371 | complement component 1, s subcomponent | NFKBIL1 | -1.80747 | nuclear factor of kappa light polypeptide gene enhancer in B-cells inhibitor-like 1 |
| CCL20 | -2.86762 | chemokine (C-C motif) ligand 20 | TLR2 | -1.69358 | toll-like receptor 2 |
| CD209 | -2.64468 | CD209 molecule | CCL4L2 | -1.57016 | chemokine (C-C motif) ligand 4-like 1; chemokine (C-C motif) ligand 4-like 2 |
| CD24 | -2.91666 | CD24 molecule; CD24 molecule-like 4 | CCL3 | -1.56671 | chemokine (C-C motif) ligand 3 |
| CD247 | -1.61306 | CD247 molecule |  |  |  |
| CD300E | -2.06229 | CD300e molecule |  |  |  |
| CD37 | -1.52292 | CD37 molecule |  |  |  |
| CD53 | -2.46814 | CD53 molecule |  |  |  |
| CD63 | -1.6236 | CD63 molecule |  |  |  |
| CD97 | -1.63984 | CD97 molecule |  |  |  |
| CFD | -1.55069 | complement factor D (adipsin) |  |  |  |
| CFH | -1.88817 | complement factor H |  |  |  |
| CNOT7 | -1.77497 | CCR4-NOT transcription complex, subunit 7 |  |  |  |
| CXCR4 | -2.84966 | chemokine (C-X-C motif) receptor 4 |  |  |  |
| HLA-DRB1 | -2.19698 | major histocompatibility complex, class II, DR beta 4; major histocompatibility complex, class II, DR beta 1 |  |  |  |
| ICAM3 | -1.77398 | intercellular adhesion molecule 3 |  |  |  |
| IFI30 | -1.82326 | interferon, gamma-inducible protein 30 |  |  |  |
| IFI35 | -2.84312 | interferon-induced protein 35 |  |  |  |
| IGHG1 | -1.74807 | immunoglobulin heavy constant gamma 1 (G1m marker) |  |  |  |
| IGSF22 | -2.61096 | immunoglobulin superfamily, member 22 |  |  |  |
| IL10RA | -1.83942 | interleukin 10 receptor, alpha |  |  |  |
| IL12RB1 | -2.77573 | interleukin 12 receptor, beta 1 |  |  |  |
| IL15RA | -1.78762 | interleukin 15 receptor, alpha |  |  |  |
| IL17RC | -2.44356 | interleukin 17 receptor C |  |  |  |
| IL1F5 | -2.26092 | interleukin 1 family, member 5 (delta) |  |  |  |
| IL28RA | -1.5629 | interleukin 28 receptor, alpha (interferon, lambda receptor) |  |  |  |
| IRF5 | -1.75591 | interferon regulatory factor 5 |  |  |  |
| ISG20L1 | -3.94781 | interferon stimulated exonuclease gene 20kDa-like 1 |  |  |  |
| ITIH5 | -1.9553 | inter-alpha (globulin) inhibitor H5 |  |  |  |
| KIR3DL1 | -2.69744 | killer cell immunoglobulin-like receptor, three domains, short cytoplasmic tail, 1; killer cell immunoglobulin-like receptor, three domains, long cytoplasmic tail, 1 |  |  |  |
| KLRC2 | -1.60524 | killer cell lectin-like receptor subfamily C, member 2 |  |  |  |
| LILRA4 | -1.64218 | leukocyte immunoglobulin-like receptor, subfamily A (with TM domain), member 4 |  |  |  |
| LST1 | -2.0501 | leukocyte specific transcript 1 |  |  |  |
| MPEG1 | -1.68675 | macrophage expressed 1 |  |  |  |
| NCF1 | -1.53641 | neutrophil cytosolic factor 1; neutrophil cytosolic factor 1C pseudogene |  |  |  |
| NCF4 | -1.92161 | neutrophil cytosolic factor 4, 40kDa |  |  |  |
| NCR2 | -1.97874 | natural cytotoxicity triggering receptor 2 |  |  |  |
| NOSIP | -3.45017 | nitric oxide synthase interacting protein |  |  |  |
| TGFA | -2.65296 | transforming growth factor, alpha |  |  |  |
| TGFB1 | -1.66731 | transforming growth factor, beta 1 |  |  |  |
| TGFB1I1 | -1.65885 | transforming growth factor beta 1 induced transcript 1 |  |  |  |
| TNFRSF4 | -1.73236 | tumor necrosis factor receptor superfamily, member 4 |  |  |  |
| TNFRSF8 | -1.71237 | tumor necrosis factor receptor superfamily, member 8 |  |  |  |
| TRAF4 | -1.83447 | TNF receptor-associated factor 4 |  |  |  |
| TRAF5 | -2.0024 | TNF receptor-associated factor 5 |  |  |  |
| TRAF7 | -1.77385 | TNF receptor-associated factor 7 |  |  |  |

Supplementary Table 1c. Apoptosis Regulation genes upregulated at 2 hrs post-stimulation.

| ***B. burgdorferi* only (2 hrs)-upregulated** | | | ***B. burgdorferi* + tick saliva (2 hrs)-upregulated** | | |
| --- | --- | --- | --- | --- | --- |
| *Gene name* | *Fold regulation* | *Gene description* | *Gene name* | *Fold regulation* | *Gene description* |
| \| **Apoptosis Regulation** \| \| --- \| | | | | | |
|  |  |  |  |  |  |
| AIFM1 | 4.513628 | apoptosis-inducing factor, mitochondrion-associated, 1 | IL19 | 1.919571 | interleukin 19 |
| BAD | 1.67231 | BCL2-associated agonist of cell death |  |  |  |
| BCL2L1 | 5.166378 | BCL2-like 1 |  |  |  |
| BCL2L13 | 6.906327 | BCL2-like 13 (apoptosis facilitator) |  |  |  |
| BCL6 | 5.479224 | B-cell CLL/lymphoma 6 |  |  |  |
| CD44 | 8.139223 | CD44 molecule (Indian blood group) |  |  |  |

Supplementary Table 1d. Apoptosis Regulation genes downregulated at 2 hrs post-stimulation.

| ***B. burgdorferi* only (2 hrs)-downregulated** | | | ***B. burgdorferi* + tick saliva (2 hrs)-downregulated** | | |
| --- | --- | --- | --- | --- | --- |
| *Gene name* | *Fold regulation* | *Gene description* | *Gene name* | *Fold regulation* | *Gene description* |
| \| **Apoptosis Regulation** \| \| --- \| | | | | | |
|  |  |  |  |  |  |
| BAG4 | -1.65192 | BCL2-associated athanogene 4 |  |  |  |
| BCL2L14 | -2.97954 | BCL2-like 14 (apoptosis facilitator) |  |  |  |
| LTBP4 | -1.84942 | latent transforming growth factor beta binding protein 4 |  |  |  |
| MAPK8 | -1.57049 | mitogen-activated protein kinase 8 |  |  |  |
| MAPK8IP2 | -2.00112 | mitogen-activated protein kinase 8 interacting protein 2 |  |  |  |
| TNFRSF4 | -1.73236 | tumor necrosis factor receptor superfamily, member 4 |  |  |  |
| TNFRSF8 | -1.71237 | tumor necrosis factor receptor superfamily, member 8 |  |  |  |
| TP53I11 | -1.59327 | tumor protein p53 inducible protein 11 |  |  |  |
| TP53I13 | -4.3537 | tumor protein p53 inducible protein 13 |  |  |  |
| TP53I3 | -1.71219 | tumor protein p53 inducible protein 3 |  |  |  |
| TRIAP1 | -2.4307 | TP53 regulated inhibitor of apoptosis 1 |  |  |  |
