# Supplementary material for: Immunomodulatory effects of tick saliva on dermal cells exposed to Borrelia burgdorferi, the agent of Lyme disease
Source: Parasit Vectors. 2016 Jul 8;9:394. doi: 10.1186/s13071-016-1638-7 (PMC4938952; doi:10.1186/s13071-016-1638-7)
Supplement: Additional file 3: Table S2. — Genes perturbed at 12 h post-stimulation. (DOCX 43 kb) [file 13071_2016_1638_MOESM3_ESM.docx]

| ***B. burgdorferi* only (12 hrs)-upregulated**  Supplementary Table 2a. Immune function genes upregulated at12 hrs post-stimulation. | | | ***B. burgdorferi* + tick saliva (12 hrs)-upregulated** | | | |
| --- | --- | --- | --- | --- | --- | --- |
| *Gene name* | *Fold regulation* | *Gene description* | | *Gene name* | *Fold regulation* | *Gene description* |
| \|  \| **Immune Function** \|  \| \| --- \| --- \| --- \| | | | | | | |
| C1QB | 1.666691 | complement component 1, q subcomponent, B chain | | CCL3 | 2.802567 | chemokine (C-C motif) ligand 3 |
| C3AR1 | 5.0202 | complement component 3a receptor 1 | | CD24 | 1.53006 | CD24 molecule; CD24 molecule-like 4 |
| CCL13 | 2.280071 | chemokine (C-C motif) ligand 13 | | CD300LB | 2.380804 | CD300 molecule-like family member b |
| CCL19 | 4.691666 | chemokine (C-C motif) ligand 19 | | CD40 | 2.368866 | CD40 molecule, TNF receptor superfamily member 5 |
| CCL20 | 7.830879 | chemokine (C-C motif) ligand 20 | | CD68 | 1.828686 |  |
| CCL4 | 2.523815 | chemokine (C-C motif) ligand 4 | | CD79B | 2.51368 | CD79b molecule, immunoglobulin-associated beta |
| CCL8 | 6.955952 | chemokine (C-C motif) ligand 8 | | HLA-DMB | 4.151252 | major histocompatibility complex, class II, DM beta |
| CCR2 | 3.044127 | chemokine (C-C motif) receptor 2 | | IL10RA | 2.531567 | interleukin 10 receptor, alpha |
| CD163 | 5.07907 | CD163 | | IL17B | 2.909046 | interleukin 17B |
| CD1A | 4.41712 | CD1A | | IL8RB | 1.951362 | interleukin 8 receptor beta |
| CD1E | 3.366696 | CD1E | | MMP25 | 1.750216 | matrix metallopeptidase 25 |
| CD300E | 5.29301 | CD300e molecule | | NFKB2 | 1.852563 | nuclear factor of kappa light polypeptide gene enhancer in B-cells 2 (p49/p100) |
| CD72 | 3.808864 | CD72 molecule | | RAC1 | 1.637092 | ras-related C3 botulinum toxin substrate 1 (rho family, small GTP binding protein Rac1) |
| CD86 | 3.352052 | CD86 molecule | | RARA | 1.593994 | retinoic acid receptor, alpha |
| CD8A | 4.043293 | CD8a molecule | | TGFB1 | 2.694657 | transforming growth factor, beta 1 |
| CDC42SE2 | 4.437 | CDC42 small effector 2 | | TNFRSF1B | 2.075066 | tumor necrosis factor receptor superfamily, member 1B |
| CFH | 4.2965 | complement factor H | | TNFSF10 | 2.416998 | tumor necrosis factor (ligand) superfamily, member 10 |
| CISH | 3.852043 | cytokine inducible SH2-containing protein | |  |  |  |
| CPLX2 | 3.516368 | complexin 2 | |  |  |  |
| CSF1 | 4.47761 | colony stimulating factor 1 (macrophage) | |  |  |  |
| CSF2RB | 4.29153 | colony stimulating factor 2 receptor, beta, low-affinity (granulocyte-macrophage) | |  |  |  |
| CXCL1 | 1.821533 | chemokine (C-X-C motif) ligand 1 (melanoma growth stimulating activity, alpha) | |  |  |  |
| CXCL13 | 7.766305 | chemokine (C-X-C motif) ligand 13 | |  |  |  |
| CXCL14 | 5.2402 | chemokine (C-X-C motif) ligand 14 | |  |  |  |
| CXCL2 | 7.906157 | chemokine (C-X-C motif) ligand 2 | |  |  |  |
| CXCL3 | 2.33577 | chemokine (C-X-C motif) ligand 3 | |  |  |  |
| CXCL6 | 8.412931 | chemokine (C-X-C motif) ligand 6 (granulocyte chemotactic protein 2) | |  |  |  |
| HLA-DQA1::HLA-DQA2 | 5.33455 |  | |  |  |  |
| HLA-DRB3::HLA-DRB4 | 5.14574 |  | |  |  |  |
| HLA-H | 4.690062 | major histocompatibility complex, class I, H (pseudogene) | |  |  |  |
| IFIH1 | 2.424118 | interferon induced with helicase C domain 1 | |  |  |  |
| IFNAR2 | 2.601607 | interferon (alpha, beta and omega) receptor 2 | |  |  |  |
| IGBP1 | 3.573158 | chromosome 14 open reading frame 19; immunoglobulin (CD79A) binding protein 1 | |  |  |  |
| IGKC | 3.6588 | immunoglobulin kappa constant; similar to Ig kappa chain V-I region HK102 precursor | |  |  |  |
| IKBKG | 4.003521 | inhibitor of kappa light polypeptide gene enhancer in B-cells, kinase gamma | |  |  |  |
| IL12B | 5.736131 | interleukin 12B (natural killer cell stimulatory factor 2, cytotoxic lymphocyte maturation factor 2, p40) | |  |  |  |
| IL12RB1 | 4.772424 | interleukin 12 receptor, beta 1 | |  |  |  |
| IL1B | 2.687375 | interleukin 1, beta | |  |  |  |
| IL1F9 | 5.062266 | interleukin 1 family, member 9 | |  |  |  |
| IL27 | 4.168173 | interleukin 27 | |  |  |  |
| IL7R | 3.6071 | interleukin 7 receptor | |  |  |  |
| IL8 | 6.148516 | interleukin 8 | |  |  |  |
| LCP1 | 4.387525 | lymphocyte cytosolic protein 1 (L-plastin) | |  |  |  |
| LILRB5 | 3.283611 | leukocyte immunoglobulin-like receptor, subfamily B (with TM and ITIM domains), member 5 | |  |  |  |
| LY6H | 4.976636 | lymphocyte antigen 6 complex, locus H | |  |  |  |
| NFKBIL1 | 4.595658 | nuclear factor of kappa light polypeptide gene enhancer in B-cells inhibitor-like 1 | |  |  |  |
| NFKBIZ | 5.115166 | nuclear factor of kappa light polypeptide gene enhancer in B-cells inhibitor, zeta | |  |  |  |
| NKTR | 1.948918 | natural killer-tumor recognition sequence | |  |  |  |
| SOCS3 | 5.439 | suppressor of cytokine signaling 3 | |  |  |  |
| SOCS5 | 2.781593 | suppressor of cytokine signaling 5 | |  |  |  |
| TIRAP | 4.082826 | toll-interleukin 1 receptor (TIR) domain containing adaptor protein | |  |  |  |
| TLR1 | 2.639102 | toll-like receptor 1 | |  |  |  |
| TLR8 | 1.633774 | toll-like receptor 8 | |  |  |  |
| TLR9 | 3.297297 | toll-like receptor 9 | |  |  |  |
| TNF | 4.472087 | tumor necrosis factor (TNF superfamily, member 2) | |  |  |  |
| TOLLIP | 5.414673 | toll interacting protein | |  |  |  |

| ***B. burgdorferi* only (12 hrs)-downregulated**  Supplementary Table 2b. Immune function genes downregulated at12 hrs post-stimulation | | | | | ***B. burgdorferi* + tick saliva (12 hrs)-downregulated** | | |
| --- | --- | --- | --- | --- | --- | --- | --- |
| *Gene name* | | *Fold regulation* | | *Gene description* | *Gene name* | *Fold regulation* | *Gene description* |
| \|  \| **Immune Function** \|  \| \| --- \| --- \| --- \| | | | | | | | |
| C1QA | -1.7348 | | complement component 1, q subcomponent, A chain | | IL13 | -4.01912 | interleukin 13 |
| C1QBP | -1.76148 | | complement component 1, q subcomponent binding protein | | IL19 | -1.93673 | interleukin 19 |
| C1QC | -1.7333 | | complement component 1, q subcomponent, C chain | | IL1RL1 | -1.58627 | interleukin 1 receptor-like 1 |
| C1RL | -2.40111 | | complement component 1, r subcomponent-like | | RAB27A | -1.80208 | RAB27A, member RAS oncogene family |
| C2 | -2.11744 | | complement component 2 | | TGFB2 | -2.40127 | transforming growth factor, beta 2 |
| CCL15 | -1.86624 | | chemokine (C-C motif) ligand 14; chemokine (C-C motif) ligand 15 | | TGFBI | -1.5119 | transforming growth factor, beta-induced, 68kDa |
| CD209 | -2.2164 | | CD209 molecule | | TNFRSF19 | -3.17125 | tumor necrosis factor receptor superfamily, member 19 |
| CD300A | -1.59552 | | CD300A molecule | | TNFRSF21 | -2.03479 | tumor necrosis factor receptor superfamily, member 21 |
| CR1 | -3.71127 | | complement component (3b/4b) receptor 1 (Knops blood group) | |  |  |  |
| DEFB103A | -2.39262 | | defensin, beta 103B; defensin, beta 103A | |  |  |  |
| EGFR | -1.69198 | | epidermal growth factor receptor | |  |  |  |
| HLA-B | -2.60055 | | major histocompatibility complex, class I, C; major histocompatibility complex, class I, B | |  |  |  |
| HLA-C | -2.73615 | | major histocompatibility complex, class I, C; major histocompatibility complex, class I, B | |  |  |  |
| HLA-DMA | -2.04452 | | major histocompatibility complex, class II, DM alpha | |  |  |  |
| HLA-DMB | -2.96443 | | major histocompatibility complex, class II, DM beta | |  |  |  |
| HLA-DPA1 | -1.94555 | | major histocompatibility complex, class II, DP alpha 1 | |  |  |  |
| HLA-DPB1 | -1.89396 | | major histocompatibility complex, class II, DQ beta 1; similar to major histocompatibility complex, class II, DQ beta 1 | |  |  |  |
| HLA-DQB2 | -1.93861 | | major histocompatibility complex, class II, DQ beta 2 | |  |  |  |
| HLA-DRB1::HLA-DRB3 | -2.48164 | | major histocompatibility complex, class II, DR beta 1 | |  |  |  |
| HLA-DRB3::HLA-DRB4 | -2.0636 | | major histocompatibility complex, class II, DR beta 3 | |  |  |  |
| HLA-E | -2.23524 | | major histocompatibility complex, class I, E | |  |  |  |
| HLA-E::LOC285831 | -2.32247 | |  | |  |  |  |
| HLA-F | -2.23726 | | major histocompatibility complex, class I, F | |  |  |  |
| HLA-H | -1.77301 | | major histocompatibility complex, class I, H (pseudogene) | |  |  |  |
| IGKC | -1.89814 | | immunoglobulin kappa constant; similar to Ig kappa chain V-I region HK102 precursor | |  |  |  |
| IGLL1 | -1.79289 | | immunoglobulin lambda-like polypeptide 1 | |  |  |  |
| IL16 | -1.95573 | | interleukin 16 (lymphocyte chemoattractant factor) | |  |  |  |
| IL17RA | -1.76503 | | interleukin 17 receptor A | |  |  |  |
| IL17RC | -2.406 | | interleukin 17 receptor C | |  |  |  |
| IL17RD | -4.1491 | | interleukin 17 receptor D | |  |  |  |
| IL21R | -1.7696 | | interleukin 21 receptor | |  |  |  |
| IL28B | -3.46589 | | interleukin 28B (interferon, lambda 3) | |  |  |  |
| IL6R | -2.26799 | | interleukin 6 receptor | |  |  |  |
| IL8RB | -3.1662 | | Interleukin 8 receptor, beta | |  |  |  |
| IRAK1 | -2.17117 | | interleukin-1 receptor-associated kinase 1 | |  |  |  |
| KIR3DL2 | -2.16119 | | killer cell immunoglobulin-like receptor | |  |  |  |
| LAG3 | -2.46394 | | lymphocyte-activation gene 3 | |  |  |  |
| LAIR1 | -1.8225 | | leukocyte-associated immunoglobulin-like receptor 1 | |  |  |  |
| LILRA6 | -2.65892 | | leukocyte immunoglobulin-like receptor, subfamily A (with TM domain), member 6 | |  |  |  |
| LILRB3 | -2.05856 | | leukocyte immunoglobulin-like receptor, subfamily B (with TM and ITIM domains), member 3 | |  |  |  |
| LILRP2 | -2.66903 | | leukocyte immunoglobulin-like receptor pseudogene 2 | |  |  |  |
| LST1 | -2.5886 | | leukocyte specific transcript 1 | |  |  |  |
| LY6G5C | -2.1679 | | lymphocyte antigen 6 complex, locus G5C | |  |  |  |
| LY6G6D | -1.83368 | | lymphocyte antigen 6 complex, locus G6F; lymphocyte antigen 6 complex, locus G6D | |  |  |  |
| LY86 | -2.06033 | | lymphocyte antigen 86 | |  |  |  |
| TCIRG1 | -2.3263 | | T-cell, immune regulator 1, ATPase, H+ transporting, lysosomal V0 subunit A3 | |  |  |  |
| TLR7 | -4.92541 | | toll-like receptor 7 | |  |  |  |
| TNFAIP8L2 | -1.89147 | | tumor necrosis factor, alpha-induced protein 8-like 2 | |  |  |  |
| TNFRSF13B | -2.09312 | | tumor necrosis factor receptor superfamily, member 13B | |  |  |  |
| TNFSF12 | -1.83172 | | TNFSF12-TNFSF13 readthrough transcript; tumor necrosis factor (ligand) superfamily, member 12; tumor necrosis factor (ligand) superfamily, member 13 | |  |  |  |
| TNFSF13 | -1.95433 | | TNFSF12-TNFSF13 readthrough transcript; tumor necrosis factor (ligand) superfamily, member 12; tumor necrosis factor (ligand) superfamily, member 13 | |  |  |  |

Supplementary Table 2c. Apoptosis regulation genes upregulated at12 hrs post-stimulation.

| ***B. burgdorferi* only (12 hrs)-upregulated** | | | | ***B. burgdorferi* + tick saliva (12 hrs)-upregulated** | | |
| --- | --- | --- | --- | --- | --- | --- |
| *Gene name* | *Fold regulation* | | *Gene description* | *Gene name* | *Fold regulation* | *Gene description* |
| \| **Apoptosis Regulation** \| \| --- \| | | | | | | |
| BCL2A1 | 2.057262 | BCL2-related protein A1 | | BAX | 2.354361 | BCL2-associated X protein |
| BCL2L11 | 2.626313 | BCL2-like 11 (apoptosis facilitator) | | CD24 | 1.53006 | CD24 molecule; CD24 molecule-like 4 |
| BNIP3 | 5.372929 | BCL2/adenovirus E1B 19kDa interacting protein 3 | | NFKBIL1 | 2.118456 | nuclear factor of kappa light polypeptide gene enhancer in B-cells inhibitor-like 1 |
| BNIP3L | 2.465026 | BCL2/adenovirus E1B 19kDa interacting protein 3-like | | RAC1 | 1.637092 | ras-related C3 botulinum toxin substrate 1 (rho family, small GTP binding protein Rac1) |
| CARD6 | 2.410432 | caspase recruitment domain family, member 6 | | RASGRF1 | 2.47314 | Ras protein-specific guanine nucleotide-releasing factor 1 |
| CASP3 | 2.750763 | caspase 3, apoptosis-related cysteine peptidase | | TGFB1 | 2.694657 | transforming growth factor, beta 1 |
| CASP4 | 3.822395 | caspase 4, apoptosis-related cysteine peptidase | | TNFAIP3 | 2.536498 | tumor necrosis factor, alpha-induced protein 3 |
| CASP5 | 5.124375 | caspase 5, apoptosis-related cysteine peptidase | |  |  |  |
| CASP6 | 1.509832 | caspase 6, apoptosis-related cysteine peptidase | |  |  |  |
| CASP7 | 3.791631 | caspase 7, apoptosis-related cysteine peptidase | |  |  |  |
| CASP8 | 5.095231 | caspase 8, apoptosis-related cysteine peptidase | |  |  |  |
| GPR65 | 2.122686 | G protein-coupled receptor 65 | |  |  |  |
| MADD | 1.592046 | MAP-kinase activating death domain | |  |  |  |
| PDCD1 | 5.015503 | programmed cell death 1 | |  |  |  |
| RASSF5 | 3.284476 | Ras association (RalGDS/AF-6) domain family member 5 | |  |  |  |
| TNFAIP8 | 3.940025 | tumor necrosis factor, alpha-induced protein 8 | |  |  |  |
| TNFRSF10B | 3.995967 | tumor necrosis factor receptor superfamily, member 10b | |  |  |  |
| TNFRSF18 | 1.561804 | tumor necrosis factor receptor superfamily, member 18 | |  |  |  |
| TNFRSF19 | 4.552765 | tumor necrosis factor receptor superfamily, member 19 | |  |  |  |
| TNFRSF1B | 1.828062 | tumor necrosis factor receptor superfamily, member 1B | |  |  |  |
| TNFRSF21 | 5.092709 | tumor necrosis factor receptor superfamily, member 21 | |  |  |  |
| TNFRSF25 | 4.2554 | tumor necrosis factor receptor superfamily, member 25 | |  |  |  |
| TNFRSF6B | 3.236997 | tumor necrosis factor receptor superfamily, member 6b, decoy; regulator of telomere elongation helicase 1 | |  |  |  |
| TNFSF10 | 4.039318 | tumor necrosis factor receptor superfamily, member 10b | |  |  |  |
| TRAF1 | 2.304555 | TNF receptor-associated factor 1 | |  |  |  |
| TRAF3 | 2.75982 | TNF receptor-associated factor 3 | |  |  |  |

Supplementary Table 2d. Apoptosis regulation genes downregulated at12 hrs post-stimulation.

| ***B. burgdorferi* only (12 hrs)-downregulated** | | | | | ***B. burgdorferi* + tick saliva (12 hrs)-downregulated** | | |
| --- | --- | --- | --- | --- | --- | --- | --- |
| *Gene name* | *Fold regulation* | | | *Gene description* | *Gene name* | *Fold regulation* | *Gene description* |
| \| **Apoptosis Regulation** \| \| --- \| | | | | | | | |
| BAD | | -1.59738 | BCL2-associated agonist of cell death | | IL13 | -4.01912 | interleukin 13 |
| BAG1 | | -2.29791 | BCL2-associated athanogene | | IL19 | -1.93673 | interleukin 19 |
| BAX | | -1.50975 | BCL2-associated X protein | | RAB27A | -1.80208 | RAB27A, member RAS oncogene family |
| BBC3 | | -2.48784 | BCL2 binding component 3 | | TGFB2 | -2.40127 | transforming growth factor, beta 2 |
| BCAP31 | | -2.20692 | B-cell receptor-associated protein 31 | | TGFBI | -1.5119 | transforming growth factor, beta-induced, 68kDa |
| BCL2 | | -2.06466 | B-cell CLL/lymphoma 2 | | TNFRSF19 | -3.17125 | tumor necrosis factor receptor superfamily, member 19 |
| BCL7C | | -2.6503 | B-cell CLL/lymphoma 7C | | TNFRSF21 | -2.03479 | tumor necrosis factor receptor superfamily, member 21 |
| BIK | | -3.26397 | BCL2-interacting killer (apoptosis-inducing) | |  | | |
| CASP2 | | -1.78565 | caspase 2, apoptosis-related cysteine peptidase | |  |  |  |
| FAF1 | | -1.72558 | Fas (TNFRSF6) associated factor 1 | |  |  |  |
| FASTK | | -2.26795 | Fas-activated serine/threonine kinase | |  |  |  |
| PDCD2 | | -1.87059 | programmed cell death 2 | |  |  |  |
| PDCD4 | | -2.64245 | programmed cell death 4 (neoplastic transformation inhibitor) | |  |  |  |
| PDCD5 | | -2.22781 | programmed cell death 5 | |  |  |  |
| TIAF1 | | -3.9368 | TGFB1-induced anti-apoptotic factor 1 | |  |  |  |
| TNFRSF25 | | -3.5203 | tumor necrosis factor receptor superfamily, member 25 | |  |  |  |
| TNFSF12 | | -1.83172 | tumor necrosis factor (ligand) superfamily, member 12, | |  |  |  |
| TNFSF13 | | -1.95433 | tumor necrosis factor (ligand) superfamily, member 13 | |  |  |  |
| TRADD | | -1.7283 | TNFRSF1A-associated via death domain | |  |  |  |
| TRAF5 | | -2.20666 | TNF receptor-associated factor 5 | |  |  |  |
| TRIAP1 | | -1.66748 | TP53 regulated inhibitor of apoptosis 1 | |  |  |  |
